# Effects of Chronic Pain Treatment on Altered Functional and Metabolic Activities in the Brain: A Systematic Review and Meta-Analysis of Functional Neuroimaging Studies

**DOI:** 10.3389/fnins.2021.684926

**Published:** 2021-07-05

**Authors:** Dongwon Kim, Younbyoung Chae, Hi-Joon Park, In-Seon Lee

**Affiliations:** ^1^College of Korean Medicine, Kyung Hee University, Seoul, South Korea; ^2^Acupuncture and Meridian Science Research Center, Kyung Hee University, Seoul, South Korea

**Keywords:** chronic pain, functional neuroimaging, activation likelihood estimation, meta-analysis, systematic review

## Abstract

Previous studies have identified altered brain changes in chronic pain patients, however, it remains unclear whether these changes are reversible. We summarized the neural and molecular changes in patients with chronic pain and employed a meta-analysis approach to quantify the changes. We included 75 studies and 11 of these 75 studies were included in the activation likelihood estimation (ALE) analysis. In the 62 functional magnetic resonance imaging (fMRI) studies, the primary somatosensory and motor cortex (SI and MI), thalamus, insula, and anterior cingulate cortex (ACC) showed significantly decreased activity after the treatments compared to baseline. In the 13 positron emission tomography (PET) studies, the SI, MI, thalamus, and insula showed significantly increased glucose uptake, blood flow, and opioid-receptor binding potentials after the treatments compared to baseline. A meta-analysis of fMRI studies in patients with chronic pain, during pain-related tasks, showed a significant deactivation likelihood cluster in the left medial posterior thalamus. Further studies are warranted to understand brain reorganization in patients with chronic pain compared to the normal state, in terms of its relationship with symptom reduction and baseline conditions.

## Introduction

Pain is a highly complex and individual experience (https://www.iasp-pain.org/), which elicits behavioral, chemical, and neuronal responses throughout the body. Under normal conditions, acute and unfamiliar pain captures our attention, and the peripheral, autonomic, and central nervous system actively respond to painful stimuli and environments (Eccleston, 1994; Basbaum et al., 2009). As the detection and modulation of pain is critical for survival, individuals have developed a pain modulation system in the body by adjusting noxious input signals and cognitive modulation of the subjective pain experience, the so-called descending pain modulatory system (Tracey and Mantyh, 2007). Unfortunately, pain could last for significantly longer and become chronic in some patients, even though the injury and painful stimulation might disappear as patients receive conventional and effective treatments.

In chronic pain conditions, defined as any pain persisting or recurring for longer than 3 months or more (Treede et al., 2015), abnormal structural, functional, and chemical changes in the periphery (e.g., sensitized peripheral receptors and nerve endings, elevated cytokine levels) (Fu et al., 2001; Wallace et al., 2001; Haroutounian et al., 2014), spinal cord (Pockett, 1995; Thomas Cheng, 2010), and brain (Apkarian et al., 2005; Smallwood et al., 2013) are related to our ability to cope with pain. A reduced threshold and excessive responsiveness to pain in the nociceptive pathway is known as central sensitization, which is a type of brain reorganization caused by neural plasticity, which leads to increased pain sensitivity (i.e., hyperalgesia) in chronic pain patients (Latremoliere and Woolf, 2009; Woolf, 2011). A significant number of studies have identified brain regions involved in the processing of acute and chronic pain, using functional neuroimaging techniques. They found an important role of the descending pain modulatory system, emotional circuitry, and learning, as well as anatomical and functional discrepancies in pain experience between healthy individuals and patients with chronic pain (Apkarian et al., 2005; Tracey and Mantyh, 2007; Henry et al., 2011; Bushnell et al., 2013). To summarize the body of evidence on the functional changes in patients with chronic pain, as compared to healthy participants, previous studies performed a meta-analysis using coordinate-based methods [e.g., activation likelihood estimation (ALE)]. For instance, Friebel et al. found that experimental painful stimulation administered to healthy participants evoked a significantly greater activation in the anterior/posterior insula, Rolandic operculum, supplementary motor area (SMA), and mid-cingulate cortex (MCC), and lesser activation in the caudal-anterior insula, anterior cingulate cortex (ACC), and supramarginal gyrus than chronic neuropathic pain conditions (Friebel et al., 2011). On the other hand, Lanz et al. found significant activation in the primary somatosensory cortex (SI), anterior/posterior insula, ACC, prefrontal cortex (PFC), thalamus, and cerebellum in healthy participants, compared to patients with neuropathic pain, and significant activation in the secondary somatosensory cortex (SII), SMA, and cerebellum in patients with neuropathic pain compared with healthy participants (Lanz et al., 2011). A more recent meta-analysis included 3,815 coordinates from functional magnetic resonance imaging (fMRI) studies applying painful cutaneous stimulation in healthy and chronic pain patients, revealing similar activation likelihood maps for healthy participants and patients with chronic pain, such as the SI, SII, thalamus, insula, cingulate cortex, PFC, basal ganglia, cerebellum, and brainstem (Tanasescu et al., 2016). The discrepant results of previous meta-analysis studies can be explained as a consequence of the heterogeneous samples and methodologies adopted across studies. Previous findings suggest that chronic pain affects the brain activity or patterns of activity in pain processing regions [which were originally referred to as the pain matrix (Talbot et al., 1991; Melzack, 2001; Duerden and Albanese, 2013)]. As chronic pain is not simply a feeling of pain for longer duration, its impact on the brain is not limited to the pain network. Instead, chronic pain is rather a complex experience which can alter many other functional networks such as corticolimbic (Baliki et al., 2012; Mansour et al., 2013), salience (Qiu et al., 2015), somatosensory-motor, and default mode networks (Baliki et al., 2008a, 2014; Becerra et al., 2014), as well as cross-network connectivity between the networks (Hemington et al., 2016; Cottam et al., 2018).

Baliki and Apkarian suggested four distinct phases of development of chronic pain: pre-injury, injury, transition, and maintenance phase, arguing that chronic pain is a neurological disease (Baliki and Apkarian, 2015). They also proposed that someone who has a cortical risk factor (e.g., lowered threshold for conscious experience of pain in response to nociceptive inputs, anatomical properties of the corticolimbic circuitry) might have a greater propensity to developing chronic pain from acute pain (Baliki et al., 2012; Mansour et al., 2013; Baliki and Apkarian, 2015; Vachon-Presseau et al., 2016b). From this point of view, we can assume that untreated changes in the brain may result in a persistent pain cycle, and that the modulation of structural and functional changes in the brain may be a key to successful chronic pain management. Growing evidence points toward the clinical effectiveness of interventions targeting brain activity in patients, such as brain stimulation, hypnosis, and meditation (Jensen M. P. et al., 2014).

A few studies have shown that the effective treatment of chronic pain reverses functional and structural brain changes in patients with chronic pain. Gwilym et al. found that decreased gray matter volume in the thalamus returned to a level similar to that in healthy volunteers after hip surgery in patients with hip osteoarthritis (OA) (Gwilym et al., 2010). Ceko et al. found that functional connectivity and white matter in the insula were normalized after treatment (spine surgery or zygapophysial joint block), and that the degree of altered functional connectivity in the insula and dorsolateral PFC was significantly correlated with pain reduction in patients with chronic low back pain (Ceko et al., 2015). In addition to the functional and anatomical changes, the effects of pain treatment on the metabolic processes in chronic pain populations have been demonstrated using Positron Emission Tomography (PET). For example, PET studies have demonstrated the effects and mechanisms of neurostimulation techniques in neuropathic pain patients. Yoon et al. found that transcranial direct current stimulation (tDCS) on the primary motor cortex (MI) in neuropathic pain patients significantly reduced subjective pain scores. They showed that glucose metabolism was significantly decreased in the dorsolateral PFC, orbitofrontal cortex (OFC), and posterior cingulate cortex (PCC), and increased in the SI, insula, and ACC in the tDCS group than in the sham stimulation group (Yoon et al., 2014). Kishima et al. also found that spinal cord stimulation significantly reduced subjective pain ratings and increased cerebral blood flow in the thalamus, MI, ACC, and dorsolateral PFC after the treatment compared to baseline (Kishima et al., 2010). These findings provide evidence for clinical use of neurostimulation treatments in chronic pain patients and insights about their actions in the brain.

It has been discussed that substantial functional, structural, and molecular changes in the brain regions and networks are associated with many chronic pain conditions, and our brain may reorganize itself after treatment, as it did when it experienced chronic pain. However, it is still unclear which regions are adaptable and which are not, or which regions are showing consistent changes across various chronic pain conditions while other regions show heterogeneous changes which cannot be detectable by meta-analytic approach. To answer this question, we reviewed previous functional neuroimaging studies that examined functional and molecular activities altered by pain relief interventions, as a whole-brain meta-analysis was carried out on a subset of the included fMRI studies that studied pain-related tasks in patient populations with chronic pain. As we assumed that imaging conditions (resting-state fMRI, task-based fMRI, PET) and tasks (pain-related, motor, cognitive, etc.) were highly heterogeneous, we limited the meta-analysis to fMRI results measured during painful tasks (painful stimulation task or pain-related motor task) with whole-brain coordinate-level data (peak x, y, z coordinates, and anatomical template).

Our research question according to the Population, Intervention, Comparator and Outcome (PICO) protocol (Higgins et al., 2019) then is: in which brain regions functional and molecular brain activities in chronic pain patients, measured by functional neuroimaging techniques such as fMRI and PET, would show consistent and significant change, either increased or decreased by treatment of chronic pain. We assumed that changes of affected functional and metabolic activities are driven by reduced pain intensity and unpleasantness in chronic pain patients, since most functional neuroimaging studies have reported significant improvements in pain and successful pain relief should be warranted for further investigation of the neural mechanisms of interventions for chronic pain. On the other hand, improvements of reward systems and cognitive networks might vary across different types of diseases and interventions as higher cognitive functions are unlikely to be changed by pain relief. Based on the assumption, we hypothesized that the treatment of chronic pain can alter functional and metabolic activities in the brain regions, especially the hub of the pain-related networks, and such changes are more consistent within the regions associated with the affective and sensory-discriminative components of pain (SI, MI, thalamus, ACC, and insula) than other regions involved in cognitive control and reward processing (limbic and frontal cortices).

## Materials and Methods

### Literature Search and Article Selection

A systematic review was conducted, following the Preferred Reporting Items for Systematic Reviews and Meta-Analyses (PRISMA) guidelines (Moher et al., 2009). Both fMRI and PET studies in chronic pain patients were searched in the PubMed and fMRI data repository NeuroSynth (neurosynth.org) on 2^nd^ February 2020. We searched PubMed using chronic pain-related keywords [chronic pain, neuropathic, chronic back pain, fibromyalgia (FM), migraine, irritable bowel syndrome (IBS), and inflammatory bowel disease], neuroimaging-related keywords (fMRI, PET, and brain activation), and treatment-related keywords (treatment, intervention, medication, drug, acupuncture, etc.). Keywords for the NeuroSynth repository search included chronic pain diseases and their relevant terms such as chronic pain, neuropathic pain, chronic back pain, chronic low back pain, FM, migraine, IBS, inflammatory bowel disease, etc., and we did not restrict the search to certain diseases. References of the searched articles were reviewed manually.

Abstracts were reviewed by two reviewers (I. S. L and D. W. K) for inclusion. We predefined eligibility criteria following the PICO protocol (Higgins et al., 2019). Articles were considered eligible if they 1) included chronic pain patients (clearly describing that patients are suffering from ‘chronic pain’ or suffering from pain disease for more than 3 months), 2) administered any type of interventions (e.g., pharmacological drug, cognitive therapy, neural stimulation), 3) included functional neuroimaging measurements (e.g., resting-state or task-related fMRI, PET), and 4) investigated patients' brain activity at least twice, before and after the treatment (henceforth referred to as PRE and POST, respectively). As we were interested in the effects of pain treatments on functional and metabolic brain activities, we excluded studies that 1) did not precisely describe the duration of pain or diagnosis criteria, 2) included chronic pain patients during a period of remission, 3) combined neuroimaging results of patients with chronic pain and other diseases, and 4) administered a treatment once inside the scanner to observe the neural responses to the treatment. We restricted our search to publications in English.

### Data Extraction

For the systematic review, we extracted the authors' name, year of publication, patient's information (disease, symptom duration, number, and age of patients), treatment, imaging condition (stimulus modality and task), and analysis method [whole-brain voxel-wise analysis, region of interest (ROI) analysis, multiple comparison correction methods, etc.]. Since the aim of this systematic review was to summarize functional changes in brain activities caused by effective treatment, we summarized treatment-induced significant changes in patients' brain functional activities and clinical outcomes, as well as reports of adverse events.

For the meta-analysis, we extracted the number of participants whose neuroimaging data were analyzed, activation (increased after the treatments in patients with chronic pain; POST > PRE) and deactivation (decreased after the treatments in patients with chronic pain; PRE > POST) coordinates, along with their associated standard anatomical template.

### Activation Likelihood Estimation Analysis

#### Whole-Brain Activation Likelihood Estimation (Pain-Related Tasks Only)

A subset of fMRI studies for the systematic review was also included in the meta-analysis using ALE analysis. In addition to the preceding inclusion criteria for the systematic review, we included all fMRI studies if peak coordinates (x, y, and z) from whole-brain volume analyses and anatomical templates for both the PRE and POST contrasts were given. Due to the high heterogeneity of tasks in task-based fMRI studies, and methodological limitations of resting-state fMRI studies, only those fMRI studies that adopted pain-related tasks were considered for the meta-analysis. Pain-related tasks were defined as any stimulations or tasks related to painful somatic sensation in patients; for example, even tactile stimuli could be included if allodynia was induced in chronic pain patients. Resting-state fMRI studies and task fMRI studies that applied pain-related emotional tasks (e.g., fear) or non-pain-related tasks (e.g., not painful tactile stimulation) were excluded from the ALE meta-analysis. The given coordinates from the included fMRI studies were used to calculate statistical maps estimating the likelihood of activation for each voxel above chance levels.

We used GingerALE (Version 3.0.2, http://brainmap.org) and performed an ALE analysis on coordinates in Montreal Neurological Institute (MNI) space (Eickhoff et al., 2012). An ALE analysis is a widely used methodology for the coordinate-based meta-analysis of neuroimaging data, in which the probability that a voxel contains at least one activation focus is calculated for each voxel by pooling the coordinates of foci reported in a series of neuroimaging studies, and convoluting a 3D Gaussian kernel by sample size to all reported foci.

All coordinates were transformed into the MNI space using the conversion tool (Talairach to MNI) implemented in the GingerALE software. Next, the included coordinates were sorted according to whether they showed either increased or decreased activation. We conducted two meta-analyses for the increased and decreased activation foci post-treatment, as compared to the pre-treatment, separately. Statistical ALE maps were corrected by cluster-based thresholding for a Family-wise error (FWE) rate corrected *P* < 0.05 at the cluster level using uncorrected *P* < 0.001 at the voxel-level as a cluster-forming threshold. To set the null distribution, 1,000 permutation tests were performed. Due to the small number of included studies, foci, and patients, we did not perform sub-group analyses.

#### Sensitivity Analysis

We performed multiple sensitivity analyses to assess the generality of the ALE analysis results. First, we repeated the ALE analysis following the removal of studies with sample size <10 to address the bias introduced by small-study effects. Additional sensitivity analyses were conducted using the leave-one-out method as multiple ALE analyses were repeatedly performed by excluding a different study each time.

## Results

### Systematic Review

**Figure 1:**
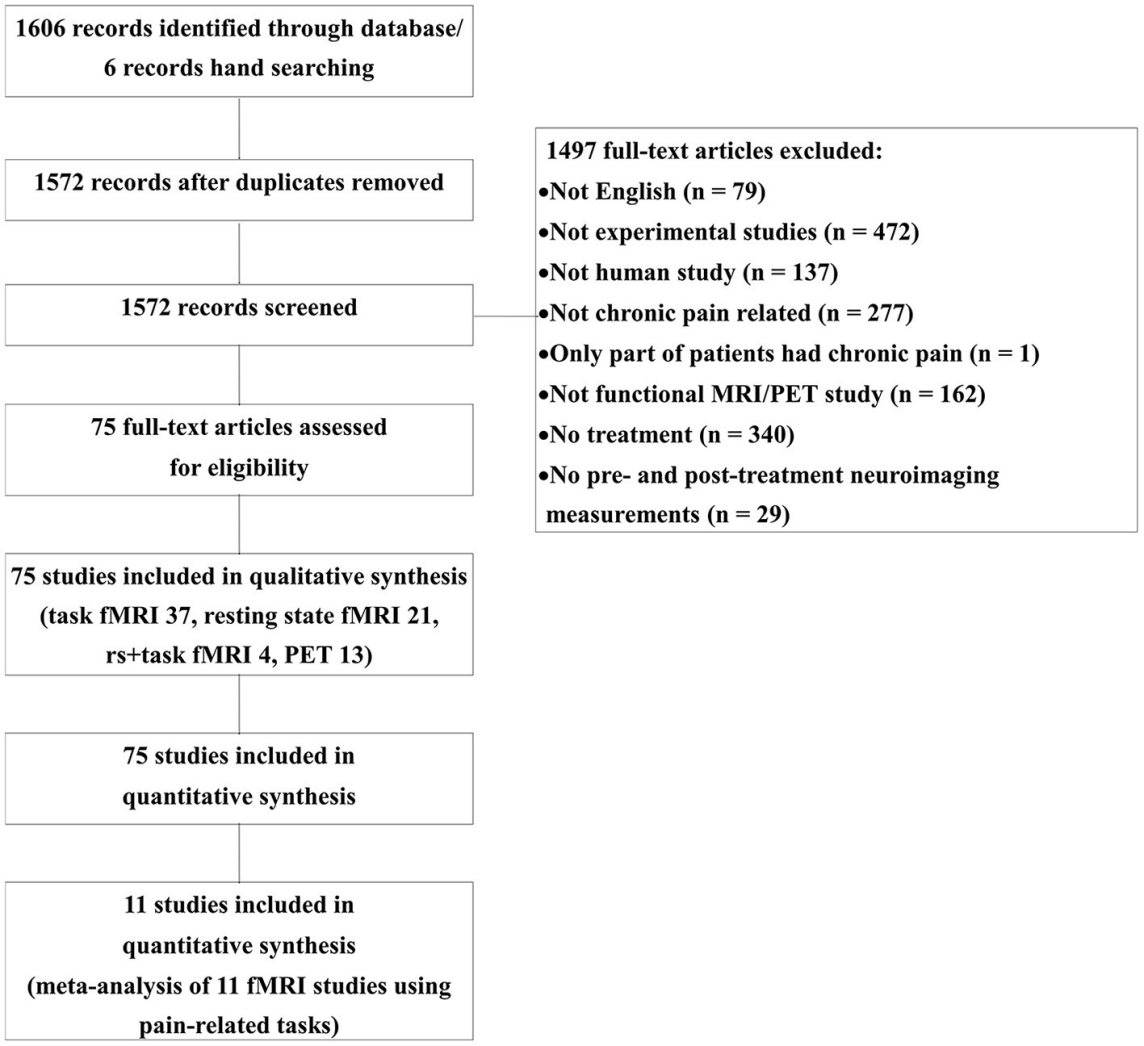
Flow diagram of literature search.

Figure 1 represents the PRISMA flow diagram of the article search (Figure 1). A total of 1,606 publications were identified through PubMed and Neurosynth, and an additional six publications were identified by manual screening. After 34 duplicates were removed, 1,497 articles were excluded. We included 75 studies in the qualitative review, and 11 of these were included in the quantitative meta-analysis. Among the 75 included studies, 13 were PET studies, 37 were task-based fMRI studies, 21 were resting-state fMRI studies, and four studies reported both resting-state activities and task-related activities. The studies were divided according to the imaging conditions, and their details are summarized in Supplementary Tables 1–4.

### Clinical Outcomes and Adverse Events

We summarized only the statistically significant clinical improvements in Supplementary Tables 1–4. Most of the studies reported significant improvements in pain response (spontaneous clinical pain, pain ratings to experimental painful stimulation, pain threshold/tolerance, pain attack time/day, tender point count, etc.) or pain-related emotional states (unpleasantness, fear, interference, etc.). In addition, symptoms unrelated to pain (e.g., defecation frequency and wrist function), depression, anxiety, quality of life, sleep quality, and medication use were evaluated using questionnaires and diaries. One study did not report any clinical outcome (Smallwood et al., 2016), and there were no significant differences in clinical outcomes between the PRE and POST visits in two studies (Petzke et al., 2013; Micalos et al., 2014). We also summarized whether they monitored and reported adverse events caused by the treatments for chronic pain. Among 75 studies, we found that 12 studies monitored and reported treatment-related adverse events (Walitt et al., 2007; Gustin et al., 2010; Tillisch et al., 2012; Petzke et al., 2013; Boyer et al., 2014; Li et al., 2015; Zhao et al., 2015, 2018; Zhang et al., 2016; Pinto-Sanchez et al., 2017; Privitera et al., 2017; Tetreault et al., 2018), and 7 studies monitored but did not report adverse events in the articles (Harris et al., 2013; Taylor et al., 2013; Jensen K. B. et al., 2014; Flodin et al., 2015; Sanders et al., 2015; Geha et al., 2016; Rogachov et al., 2019).

### Brain Changes by Treatment of Chronic Pain (fMRI Studies)

The fMRI studies included in the present systematic review have been summarized in Supplementary Tables 1–3. Chronic pain conditions included FM (*n* = 15), neuropathic pain (post-stroke central pain, post-herpetic neuralgia, CRPS, cervical spondylosis neck pain, carpal tunnel syndrome, and amputation/phantom limb pain; *n* = 10), chronic low back pain (*n* = 9), headache/migraine (*n* = 8), chronic OA (knee or hand), IBS (*n* = 7), chronic musculoskeletal pain, somatoform pain disorder, Crohn's disease, inflammatory bowel disease, erythromelalgia, and failed back surgery syndrome (*n* = 1).

The interventions employed in the studies included pharmacological treatments (sphenopalatine ganglion blockade, pregabalin, duloxetine, ketamine, lidocaine patch, etc.; *n* = 23), acupuncture or electro-acupuncture (*n* = 18), behavioral/cognitive/education/exercise therapy (*n* = 12), neurostimulation [tDCS, transcranial magnetic stimulation (TMS), and spinal cord stimulation; *n* = 5], surgery or invasive intervention (*n* = 3), drug withdrawal, and moxibustion (*n* = 2).

For the task-based fMRI studies, 27 studies employed pain-related tasks such as spontaneous pain rating, receiving painful stimulation, and hand squeezing, while another 10 studies used non-pain-related tasks such as visual tasks and non-painful tactile stimulation during fMRI scanning. The most common analysis methods were ROI-based analyses (*n* = 20) and correlation analyses (mostly ROI-based correlation analysis between brain activity and clinical outcomes; *n* = 22). Only a few studies applied a functional connectivity analysis (Napadow et al., 2007; Hashmi et al., 2012), principal component analysis, spectral power analysis (Hashmi et al., 2012), and covariate analysis (Geha et al., 2007; Baliki et al., 2008b; Parks et al., 2011). For the analysis of resting-state fMRI, a functional connectivity analysis (*n* = 16) and independent component analysis (ICA; *n* = 14) were the most popular approaches, and amplitude of low frequency fluctuations (ALFF) or fractional ALFF (Shpaner et al., 2014; Li et al., 2017), multi-voxel pattern analysis/machine learning (Chen et al., 2018; Rogachov et al., 2019) (*n* = 2), graph (Tetreault et al., 2018), and regional homogeneity analysis (Chen et al., 2018) (*n* = 1) were also used. For the ICA, the default mode network was the most frequently targeted functional network (Napadow et al., 2012; Li et al., 2014; Shpaner et al., 2014; Yoshino et al., 2018; Rogachov et al., 2019; Zou et al., 2019) (*n* = 5); the salience, basal ganglia, fronto-parietal, sensorimotor, central-executive, and dorsal attention networks were also targeted.

The summarized results of the PRE vs. POST contrasts of fMRI studies in the pain processing regions are shown in Table 1. The SI (7 of 9 studies), MI (4 of 5 studies), and thalamus (5 of 6 studies) showed significantly decreased activity after treatment compared to the pre-treatment baseline. The ACC (increased *n* = 3, decreased n = 6) and insula (increased *n* = 5, decreased *n* = 8) showed a trend of decrease. On the other hand, the trend of increase or decrease was not clear for the SII (increased *n* = 2, decreased *n* = 2), PCC (increased *n* = 3, decreased *n* = 3), PFC (increased *n* = 3, decreased *n* = 5), and OFC (increased *n* = 2, decreased *n* = 1).

**Table 1:** Summary of changes in functional activities (pre-treatment vs. post-treatment) in the fMRI studies.

**References (task)**	**Chronic pain**	**Treatment (duration)**	**SI**	**THAL**	**MI**	**INS**	**ACC**	**PCC**	**PFC**	**OFC**	**SII/OPER**
Jensen et al. (2012) (pain)	FM	CBT (12 w)				**↑**			**↑**	**↑**	
Kim et al. (2013) (pain)	FM	Pregabaline	**↓**	**↓**		**↓**					
Koeppe et al. (2004) (pain)	FM	Tropisetron/prilocaine	**↓**			**↓**	**↓**				
Petzke et al. (2013) (pain)	FM	Milnacipran	**↑**	**↑**		**↑**	**↑**	**↑**			
Taylor et al. (2013) (pain)	FM	Cranial electrical stimulation (8 w)		**↓**		**↑**	**↓**	**↓**	**↑**		
Harte et al. (2016) (visual)	FM	Pregabalin (17 days)				**↓**					
Harris et al. (2013) (pain)	FM	Pregabalin (2 w)									
Baliki et al. (2008b) (pain)	LBP	lidocaine patch (2 w)		**↓**		**↓**	**↓**		**↓**		
Smallwood et al. (2016) (pain)	LBP	Acceptance-commitment therapy/health education program (4 w)				**↓**	**↓**	**↓**			
Seminowicz et al. (2011) (MSIT)	LBP	Spine surgery or zygapophysial joint injections							**↓**		
Timmers et al. (2019) (visual)	LBP	Exposure therapy	**↓**		**↓**				**↓**		
Sanders et al. (2015) (pain)	Hand OA	Naproxen (1 w)	**↓**	**↓**	**↓**	**↓**		**↓**			
Shpaner et al. (2014) (REST)	Musculoskeletal pain	CBT (11 w)						**↑**			
Chen et al. (2015) (ACU)	Knee OA	ACU (4 w)									**↑**
Gustin et al. (2010) (pain)	Neuropathic pain (CRPS)	NMDA-REC antagonist, morphine (7–8 w)	**↓**				**↓**				
Geha et al. (2007) (pain)	Neuropathic pain (post-herpetic neuralgia)	Lidocaine (2 w)	**↓**	**↓**	**↓**	**↑**				**↓**	**↓**
Napadow et al. (2007) (ACU)	Neuropathic pain (CTS)	ACU (5 w)	**↑**			**↑**	**↑**		**↑**		
Grazzi et al. (2010) (pain)	Migraine with medication overuse	Drug withdrawal (6 m)				**↓**			**↓**		**↓**
Li et al. (2017) (REST)	Migraine without aura	ACU (4 w)								**↑**	
Chu et al. (2012) (pain)	IBS	EA			**↑**						**↑**
Zhao et al. (2018) (pain)	IBS	EA/moxibustion (4 w)				**↓**	**↓**		**↓**		
Geha et al. (2016) (pain)	Erythromelalgia	Carbamazepine (4 w)	**↓**		**↓**		**↑**	**↑**			

*Red cells with up-arrow indicate increased functional brain activities after the treatments compared to those before the treatments of chronic pain (POST > PRE). Blue cells with down-arrow indicate decreased functional brain activities after the treatments compared to those before the treatments of chronic pain (PRE > POST). ACU, acupuncture; ACC, anterior cingulate cortex; CBT, cognitive behavioral therapy; CRPS, complex regional pain syndrome; CTS, carpal tunnel syndrome; EA, electro-acupuncture; FM, fibromyalgia; INS, insula; LBP, low back pain; m, months; MI, primary motor cortex; MSIT, multi-source interference task; NMDA, N-methyl-D-aspartate; OA, osteoarthritis; OFC, orbitofrontal cortex; OPER, Rolandic operculum; PCC, posterior cingulate cortex; PFC, prefrontal cortex; REC, receptor; REST, resting-state; SI, primary somatosensory cortex; SII, secondary somatosensory cortex; THAL, thalamus; w, week(s)*.

### Brain Changes by Treatment of Chronic Pain (PET Studies)

A summary of the PET studies used in the present systematic review is presented in Supplementary Table 4. Patients with FM (Walitt et al., 2007; Harris et al., 2009; Boyer et al., 2014; Sawaddiruk et al., 2019), IBS (Berman et al., 2002; Mayer et al., 2002; Lieberman et al., 2004; Lackner et al., 2006) (*n* = 4), neuropathic pain (Maarrawi et al., 2007; Kishima et al., 2010; Yoon et al., 2014) (*n* = 3), headache (Magis et al., 2011), and chronic tennis elbow (Linnman et al., 2016) (*n* = 1) were included in the PET imaging studies. To treat chronic pain patients, neurostimulation (occipital nerve, spinal cord, tDCS/TMS, etc.; *n* = 5), pharmacological treatment (alosetron, pregabalin, Coenzyme Q10; *n* = 3), cognitive/exercise therapy (*n* = 2), acupuncture, individualized combination therapy, and placebo regimen (*n* = 1) were administered to patients with chronic pain. Using PET imaging technology, patients' glucose uptake, blood flow (*n* = 5), and opioid (*n* = 2) and neurokinin 1 receptor binding (*n* = 1) in the brain tissue were measured before and after the treatments. Positron emission tomography imaging scans were conducted during the rest (*n* = 8), visceral distention (*n* = 4), and treatment interventions (occipital neurostimulation and acupuncture; *n* = 1, respectively). Whole-brain analysis (*n* = 12), ROI-based analysis (*n* = 8), and correlation analysis (*n* = 4) were the most popular analysis methods for PET imaging studies.

A summary of PRE vs. POST results of PET studies, in which either the whole-brain or regions (volumes) of interest analysis was used, in the pain processing regions is presented in Table 2. The SI, MI, thalamus, and insula showed significantly increased tissue glucose uptake, blood flow, and μ-opioid receptor binding potentials after the treatments, compared to the pre-treatment baseline, while PCC showed significantly decreased glucose uptake and blood flow. Two PET studies showed that the blood flow was increased in the insula, and decreased in the ACC, in response to painful stimulation after the treatment compared to the baseline (Berman et al., 2002; Mayer et al., 2002). The SII did not show significant changes between the PRE and POST conditions in PET imaging studies.

**Table 2:** Summary of metabolic changes (pre-treatment vs. post-treatment) in the PET studies.

**References (task, analysis)**	**Chronic pain**	**Treatment (duration)**	**SI**	**THAL**	**MI**	**INS**	**ACC**	**PCC**	**PFC**	**OFC**	**SII/ OPER**
Walitt et al. (2007) (REST, VOIs)	FM	Individualized, comprehensive (8 w)		**Glu** **↑**		**Glu** **↑**				**Glu** **↑**	
Magis et al. (2011) (occipital nerve stimulation, ROIs)	Drug-resistant chronic cluster headache	Occipital nerve stimulation (various)	**Glu** **↑**		**Glu** **↑**		**Glu** **↓**				
Yoon et al. (2014) (REST, whole-brain)	Neuropathic pain	tDCS on the MI (10 days)	**Glu** **↑**	**Glu** **↓**		**Glu** **↑**	**Glu** **↑**	**Glu** **↓**	**Glu** **↓**	**Glu** **↓**	
Berman et al. (2002) (pain, whole-brain, ROIs)	IBS	Alosteron (3 w)				**Blood flow** **↑**	**Blood flow** **↓**				
Mayer et al. (2002) (pain, ROIs)	IBS	Alosteron (3 w)				**Blood flow** **↑**	**Blood flow** **↓**		**Blood flow** **↑**	**Blood flow** **↓**	
Lackner et al. (2006) (REST, whole-brain)	IBS	Cognitive therapy (10 w)					**Blood flow** **↓**	**Blood flow** **↓**			
Kishima et al. (2010) (REST, whole-brain)	Neuropathic pain	Spinal cord stimulation (>6 m)		**Blood flow** **↑**	**Blood flow** **↑**		**Blood flow** **↑**		**Blood flow** **↑**	**Blood flow** **↑**	
Maarrawi et al. (2007) (REST, VOIs)	Neuropathic pain	Motor cortex stimulation (7 m)							**Opioid REC** **↓**		
Harris et al. (2009) (ACU, ROIs)	FM	ACU (4 w)		**μ-opioid REC** **↑**		**μ-opioid REC** **↑**	**μ-opioid REC** **↑**				

*Red cells with up-arrow indicate increased measures of PET scans (e.g., glucose uptake, receptor bindings, etc.) after the treatments compared to those before the treatments of chronic pain (POST > PRE). Blue cells with down-arrow indicate decreased measured PET scans after the treatments compared to those before the treatments of chronic pain (PRE > POST). ACU, acupuncture; ACC, anterior cingulate cortex; FM, fibromyalgia; Glu, glucose; IBS, irritable bowel syndrome; INS, insula; LBP, low back pain; m, months; the MI, primary motor cortex; OFC, orbitofrontal cortex; OPER, Rolandic operculum; PCC, posterior cingulate cortex; PFC, prefrontal cortex; REC, receptor; REST, resting-state; ROIs, regions of interest; SI, primary somatosensory cortex; SII, secondary somatosensory cortex; tDCS, transcranial direct current stimulation; THAL, thalamus; VOIs, volumes of interest; w, week(s)*.

### Whole-Brain ALE Meta-Analysis of Pain-Related Task fMRI Studies

#### Significant Clusters for After Treatment vs. Baseline Contrast During Pain Tasks

For the coordinate based meta-analysis of whole-brain fMRI studies, 118 activation and deactivation foci were extracted from 11 pain-related fMRI studies in 256 chronic pain patients (222 female). Chronic pain conditions included FM (*n* = 4), neuropathic pain (post-herpetic neuralgia, CRPS; *n* = 2), chronic OA (knee or hand; *n* = 2), chronic low back pain, irritable bowel disease, and erythromelalgia (*n* = 1). Since the aim of the coordinate-based ALE analysis was to identify brain regions that are significantly modulated by pain treatments, we included the significantly activated or deactivated foci separately during the pain tasks extracted from the PRE vs. POST contrasts. The pain-related tasks in fMRI studies included in the meta-analysis were spontaneous pain rating, movement of the paining body parts (e.g., hand squeezing), and receiving painful stimulation tasks (painful thermal, cold, and mechanical stimulation including rectal distention).

**Figure 2:**
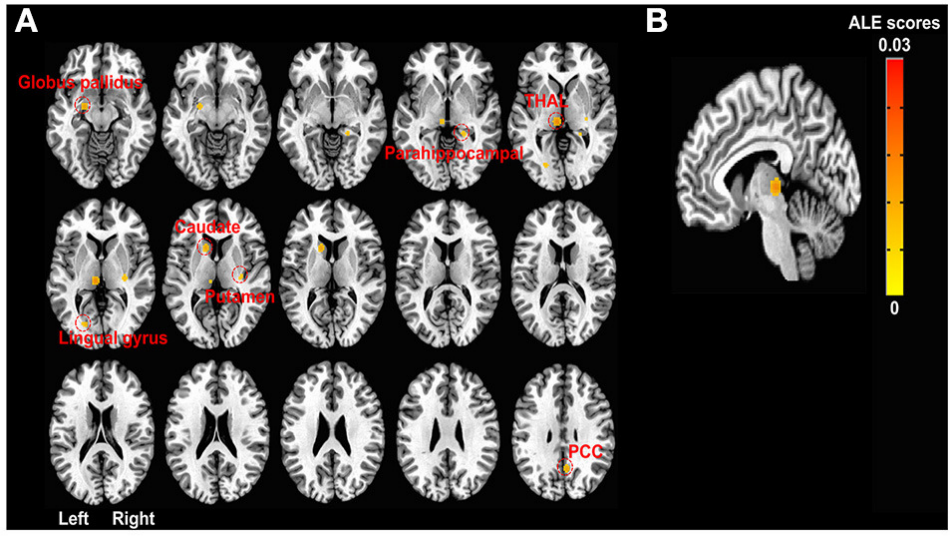
ALE meta-analyses of fMRI studies in chronic pain patients during pain-related tasks. **(A)** Activation likelihood map showing seven deactivation clusters at uncorrected *p* < 0.001 and extent threshold *k* > 30. Deactivation clusters include the left thalamus (−6, 24, 0; 99 voxels), caudate (−14, −14, 8; 55 voxels), globus pallidus (−20, 6, −12; 51 voxels), lingual gyrus (−20, 76, 2; 31 voxels), and right parahippocampal gyrus (22, 38, −4; 30 voxels), putamen (30, 22, 2; 36 voxels), and posterior cingulate gyrus (6, 58, 32; 52 voxels) after the chronic pain treatments compared to baseline (before the treatments) while patients with chronic pain were performing pain-related tasks (e.g., hand squeezing) or receiving painful stimulation. All clusters of activation (increased activation likelihood scores after pain treatments than before the treatments) at the uncorrected threshold were smaller than the minimum cluster size (*k* > 30). **(B)** FWE rate corrected activation likelihood map showing the significant deactivation cluster in the left medial posterior thalamus (−6, 24, 0; 99 voxels) after the chronic pain treatments compared to the baseline (before the treatments) while chronic pain patients were performing pain-related tasks (e.g., hand squeezing) or receiving painful stimulation (*P* < 0.05 at cluster-level and uncorrected *P* < 0.001 at voxel-level, *k* > 30). Seven out of ten leave-one-out sensitivity analyses also showed a significantly decreased likelihood of activation in the left thalamus (FWE corrected as above, cluster sizes range 99–100). No clusters of activation passed the multiple comparison correction threshold. ALE, activation likelihood estimation; FWE, Family-wise error; PCC, posterior cingulate cortex; THAL, thalamus.

There were no significantly activated foci that survived a multiple comparison correction (PRE < POST during pain tasks). The likelihood of deactivation was significant after a multiple comparison correction in the left medial posterior thalamus (*x* = −6, *y* = 24, *z* = 0; *p* < 0.001, Figure 2B), pointing thereby to a significant probability of deactivation in the medial posterior thalamus in response to pain-related tasks after the treatment compared to the pre-treatment states (POST < PRE during pain tasks). The left thalamus, caudate, globus pallidus, BA 18 (lingual gyrus), and right BA 30 (parahippocampal gyrus), putamen, and BA 31 (cingulate gyrus) were also deactivated at uncorrected *p* < 0.001 and extent threshold *k* > 30 (Figure 2A).

#### Sensitivity Analysis

Four studies had small sample sizes (*n* < 10) (Koeppe et al., 2004; Baliki et al., 2008b; Kim et al., 2013; Geha et al., 2016), and the results of the ALE analysis after removal of these studies did not survive FWE correction (*P* < 0.05 at cluster-level and uncorrected *P* < 0.001 at voxel-level, *k* > 30). According to the leave-one-out sensitivity analysis, 7 out of 10 ALE analyses on coordinates of deactivation foci showed a significantly decreased likelihood of activation in the left thalamus (FWE corrected as above, cluster sizes range 99–100). No activation foci survived a multiple comparison correction in any of the leave-one-out sensitivity analyses.

## Discussion

Becerra et al. defined the effect of treatment by comparing brain activities in patients with severe pain before treatment with those experiencing less pain after treatment. They also depicted the residual effect after treatment, which means the remaining differences in neural properties between patients with chronic pain and healthy controls (Becerra et al., 2014). To the best of our knowledge, this is the first systematic review and meta-analysis study to determine which brain regions show changes in their functions and metabolic states (the treatment effect), and which regions show remaining alterations (the residual effect) after chronic pain treatment.

In the neuroimaging studies comparing pre- and post-treatment conditions, task-based fMRI is the most frequently applied imaging technique, followed by resting-state fMRI scanning and PET imaging. In addition to the significant improvements in pain intensity or pain-related emotional states, psychological states (depression, anxiety), quality of life, and medication use were also evaluated. Among 75 studies, only 12 studies monitored and reported treatment-related adverse events. A meta-analysis of clinical outcomes was not performed since there were only few randomized controlled trials, and the data necessary to determine the effect size was not reported in most of the studies. Although clinical neuroimaging studies have been focusing on the changes of brain activities, however, we argue that we should improve the quality of reporting of clinical and behavioral data including adverse events. For example, there are discrepancies between samples from whom neuroimaging and clinical data were collected. Clinical outcomes were often measured using different types of questionnaires or scales, although heterogeneity of the included studies also contributed to the heterogeneous clinical measurements, and it makes us impossible to quantitatively summarize the results across the studies. Although only a few studies provided relevant data, we assessed the publication bias and heterogeneity using available data from 11 studies and found that there's a significant positive publication bias (Egger's regression *t* = 11.08, *p* < 0.001) and moderate heterogeneity (*I*^2^ = 58.73%). They suggest that the intervention effects were overestimated or there might be a positive reporting bias, and caution is needed in interpreting clinical results of the neuroimaging studies due to an insufficient number of treatment sessions and lack of an appropriate control group or intervention.

We found a tendency of decreased functional activities (in the SI, MI, thalamus, insula, and ACC) and increased molecular activities in the sensory-discriminative and affective regions (glucose consumption in the SI, glucose consumption and blood flow in the MI, glucose consumption, blood flow, and μ-opioid receptor binding potential in the thalamus and insula) after the treatments than before the treatments of chronic pain, while other regions involved in reward and cognitive processes (amygdala, PFC, OFC, etc.) yielded inconsistent results across the studies. If we assume that these changes are associated with pain relief, decreased functional activities might indicate recovery from the sensitized response to pain-related tasks by the treatments. On the other hand, molecular activities in the SI, MI, thalamus, and insula might be elevated during the resting and pain conditions. The results suggest that successful pain treatments reduced processing of pain-related information and boosted the metabolic functions of the brain, so that the altered functional activities could be reversed while processing sensory-discriminative and affective components of pain. On the other hand, the regions involved in cognitive execution and reward processing functions might be less adjustable in response to pain treatments, as the complicated and higher cognitive functions might be treated neither consistently nor completely in the included studies. Further studies would be required to substantiate this inference.

The multiple-comparison-corrected ALE analysis showed a significantly decreased likelihood of activation in the left medial posterior thalamus during pain-related tasks in chronic pain patients after treatment than before treatment (the treatment effect), which suggests that the thalamus, especially the medial posterior part, is the most consistent region affected by pain treatments in patients with chronic pain. The thalamus is the transmission center of acute noxious signals from peripheral receptors to the cortex (e.g., SI and insula) through spinothalamic track, and also plays an important role in pain modulation and chronification. Pain modulatory effects of thalamus stimulation have been tested since the 1970s (Hosobuchi et al., 1973; Mayer and Liebeskind, 1974; Mazars, 1975; Rhodes and Liebeskind, 1978; Kupers et al., 2000), and thalamic stimulation influences the cerebral blood flow in the insula (Duncan et al., 1998), PAG, PFC (Kupers et al., 2000), and ACC (Davis et al., 2000). According to previous meta-analyses, the evidence of functional activities in the thalamus in patients with chronic pain compared to healthy controls is conflicting. Lanz et al. (2011) and Jensen et al. (2016) found that the thalamus was activated by pain tasks in both healthy and affected patients, and the healthy controls showed significantly stronger activity in the thalamus compared to the patients during pain experience. They considered, as such, that decreased activation likelihood in the thalamus may support decreased sensory processing in patients with chronic pain due to the continuous noxious input to the brain, and thalamic disturbances may lead to chronic pain symptoms. Since not all fMRI studies that reported PRE vs. POST contrasts reported them separately for patients and control groups, we could not conclude whether the decreased activation likelihood in the thalamus after the treatments is due to the increased activity in the thalamus during pain processing before the treatments (as we discussed above) or not. We also cannot neglect the fact that functional activities were actually decreased in the thalamus during pain processing in patients with chronic pain than in healthy controls, as shown in a previous meta-analysis, and that these activities might be decreased further after treatment, as our study showed. If so, it suggests that the functional activities in the thalamus may not return to a normal state and may only change upon treatment to a new state.

The thalamus, ACC, and insula constitute the salience and central autonomic network as well as the pain network (Lee et al., 2020), and they are also commonly activated during tasks related to non-painful stimulation, emotion, memory, and interoception (Cauda et al., 2012). Thus, decreased likelihood of activation in the thalamus and a tendency of decreased functional activities in the insula and ACC might be not solely related to the reduced pain sensations, but they might be also associated with the reduced salience processing. As there is an active debate about the pain specificity in the brain, please see Legrain et al. (2011), Mouraux and Iannetti (2018), and Lee et al. (2020) for more discussion.

Growing evidence demonstrates that chronic pain is not a longer-lasting form of acute pain. Rather, chronic pain has distinct mechanisms and mediators than acute pain in healthy population, albeit with some overlap, and various factors contribute to chronic pain such as psychological (Vachon-Presseau et al., 2016a; Hruschak and Cochran, 2018), molecular, structural, and functional neural mechanisms (Davis and Moayedi, 2013; Bliss et al., 2016; Taylor et al., 2016; Groh et al., 2018; Adebiyi et al., 2019). It has been argued that chronic pain is a disease, in particular, a brain disease (Tracey and Bushnell, 2009; Davis and Moayedi, 2013), and not just a pain symptom left untreated or under-treated (Raffaeli and Arnaudo, 2017) [see Cohen et al. (2013) and Sullivan et al. (2013) for the opposite opinions, and it is noteworthy that we should also consider various chronic pain subgroups when dealing with this issue (Treede et al., 2019)]. If untreated changes in the brain result in a persistent pain cycle, the reversal of altered brain structural and functional changes toward a normal state may contribute to the relief of chronic pain. It is also plausible that chronic pain relief can normalize structural, functional, and metabolic abnormalities in the brain. Although we cannot assess the direction of effects between “normalized brain” and “reduced pain” in this study, this will be an interesting topic for further research to estimate the influence of pain reduction over brain changes, and the influence of the brain alterations on symptom improvement.

A survey of patients with chronic pain in Europe showed that one-third of the patients were currently not being treated; only 2% of them were currently treated by a pain specialist, and 40% were receiving inadequate pain management (Breivik et al., 2006). Moreover, oral analgesics, one of the fastest and cheapest medications for pain treatment, are not suitable for long-term pain relief due to side effects, and patients' tolerance to opioids is increasing, which means that the dose needs to be increased over time to achieve a certain amount of pain relief (Hylands-White et al., 2017). Recently, chronic pain treatment, which targets neural abnormalities in patients, has been receiving more attention (Borsook et al., 2007; Bentley et al., 2016) along with the advancement of pharmacological fMRI (investigating the effects of pharmacological interventions on functional brain changes) (Duff et al., 2015; Bauch et al., 2017) and increasing efforts to search for brain markers for pain in the brain (Wager et al., 2013; Duff et al., 2015; Lopez-Sola et al., 2017). Although this study only covers central functional and metabolic changes in chronic patients, our approach is important for identifying neural targets for chronic pain treatment. Identifying brain regions responding to the treatment offers valuable insight for the development of neural markers of diseases (e.g., markers for disease and symptom severity) and treatment effects (e.g., markers for symptom improvement of a certain treatment). For example, μ-opioid receptor binding potentials were significantly increased in the dorsolateral PFC, ACC, thalamus, amygdala, putamen, and nucleus accumbens after treatment than before treatment, and binding potentials were significantly correlated with clinical pain ratings (Harris et al., 2009), which implies that the μ-opioid receptor binding potentials are a strong candidate for chronic pain markers. In addition, intervention-related and disease-related functional and metabolic changes induced by intervention in a single subject will contribute to personalized pain management, as pain is an individual experience originating from the neuropathological state of the brain. We can estimate the effect of chronic pain in the brain, and also estimate and re-evaluate the treatment effects focusing on neural changes using neuroimaging techniques. If we combine multiple neuroimaging techniques and measure various changes in the brain, such as structural, functional, and metabolic changes, we could develop a marker for chronic pain as well as a marker for the treatment effect, in the future. Questions related to future pain management will include “which treatment is more effective in alleviating hyper-sensitized thalamic response to painful stimulation?,” “where should we target in the brain to reduce spontaneous pain?,” and “which chronic pain disease is more likely to be treated by central manipulation (e.g., TMS) on the motor cortex?”; our approach may provide preliminary answers to the questions.

This study has a number of limitations. As we strictly restricted our analysis to the effects of chronic pain interventions, and did not include functional neuroimaging studies that did not involve chronic pain treatment, only a small number of studies were included in our ALE meta-analysis. A cautious interpretation of the results of the present study is necessary as underpowered studies have increased risk of false positives (Button et al., 2013). Previous meta-analysis studies may be referred to with regard to brain activation during painful stimulation in patients with chronic pain, irrelevant to the effect of treatment, corresponding (but not equivalent) to the PRE contrast in this study (Friebel et al., 2011; Lanz et al., 2011; Tanasescu et al., 2016). As participants of included studies had different diseases with different pathophysiology, our results need further validation. Moreover, they were treated by various interventions and few studies had small sample sizes, and most of the included studies were carried out only in adult populations (over 20 years old). All of these restrict generalizability of the results. Thus, we are not arguing that all chronic pain diseases have a single common mechanism, or that we could generalize the results to a larger population or apply them to a specific disease and intervention. Instead, our finding suggests that there might be a hub region in the brain which remains malleable in response to pain treatment, so that it can adapt itself to changes in the pain state. We need further investigation to clarify whether these changes are causally induced by pain relief or not, and whether the changes we found are similar or different across pain conditions. Moreover, due to methodological constraints, we were not able to measure the relationship between the degree of the treatment effect (the amount of pain reduction) and the degree of functional and metabolic changes in the brain. A meta-analysis of resting-state fMRI studies was technically available (Zang et al., 2015); however, we did not conduct the meta-analysis by combining heterogeneous statistical outcomes (ALFF, regional homogeneity, functional connectivity, etc.) as it might provide false information.

Based on the limitations of our study, we make following suggestions which can benefit future research. We suggest that clinical changes and adverse events should be monitored and reported in detail in future studies, as treatments should be effective and safe, and functional neuroimaging studies are no exception to this point. To analyze the relationship between the treatments and outcomes (e.g., pain intensity, pain interference, psychological state, quality of life, etc.) accurately, we need a standard guideline for reporting clinical outcomes in neuroimaging studies. Lastly, future study should further investigate “how” and “which factors” facilitate the changes of functional and molecular neural activities in chronic pain patients after treatment.

## Conclusion

A systematic review of 62 fMRI and 13 PET studies showed the functional (decreased functional activities) and metabolic changes (increased glucose uptake, blood flow, μ-opioid receptor binding potentials) in the somatosensory-discriminative regions (SI and thalamus) and the MI after the treatment of chronic pain compared to the pre-treatment baseline. Meta-analysis of 11 pain-related task fMRI studies showed that the activation likelihood in the thalamus was significantly decreased after the treatment compared to before the treatment. Our findings might help us develop a new treatment for chronic pain management that focuses on neural reorganization and allow for personalized treatment of chronic pain by considering disease-specific, intervention-specific, and individual-specific responses in patients with chronic pain in the future.

## Data Availability Statement

Data are available upon request to the corresponding author.

## Author Contributions

DK and I-SL carried out the data extraction and meta-analysis, prepared the first draft. YC and H-JP interpreted the data. I-SL finalized the manuscript. YC, H-JP, and I-SL performed the critical revision of the manuscript for important intellectual content. All authors contributed to the article and approved the submitted version.

## Conflict of Interest

The authors declare that the research was conducted in the absence of any commercial or financial relationships that could be construed as a potential conflict of interest.
