# Supplementary material for: Effects of Chronic Pain Treatment on Altered Functional and Metabolic Activities in the Brain: A Systematic Review and Meta-Analysis of Functional Neuroimaging Studies
Source: Front Neurosci. 2021 Jul 5;15:684926. doi: 10.3389/fnins.2021.684926 (PMC8287208; doi:10.3389/fnins.2021.684926)
Supplement: Supplementary file 1 [file Table_1.docx]

**Supplementary table 1. Task fMRI studies**

| ***No. Author, year (study type)** | **Patients, disease duration, No., mean age±SD** | **Treatment (no, duration)** | **MRI, stimulation and task, standard template, correction** | **Contrasts for voxel-wise analysis** | **Other analyses** | **PRE vs. POST in the brain** | **PRE vs. POST of clinical/behavioral outcomes** | **Adverse events** |
| --- | --- | --- | --- | --- | --- | --- | --- | --- |
|  |  |  |  |  | **ROIs** |  |  |  |
| **Pain-related task studies** | | | | | | | | |
| ***1. Koeppe, 2004 (RCT)[33]** | **FM** (6; **4** received tropisetron, all F; 56±7) | 5-HT3 receptor antagonist tropisetron/prilocaine (2 times, 5 d) | 1.5T, painful stimulation (pressure), TAL, Yes | PRE vs. POST | - | Decrease: SI, post. INS,  ACC | Decreased: stimulus unpleasantness | - |
| ***2. Geha, 2007 (CT)[18]** | **Post-herpetic neuralgia** > 3 mon (14; **11** analyzed, 10 F; 67.8) | Lidocaine patch (2 w) | 3T, spontaneous pain rating/visual, MNI, Yes | PRE,  POST1 (6 h after Tx), POST2 (2 w after Tx), PRE + POST1 + POST2, monotonic decrease with sessions | Covariate, correlation | Decreased: THAL, hypoTHAL, SI, MI, SII, INS, ventral striatum, preCUN, OFC | Decreased: spontaneous pain, NPS | - |
|  |  |  |  |  |  | Increased: inf. frontal, ant. INS |  |  |
| ***3. Baliki, 2008 (CT)[1]** | **CLBP** > 3 mon (11; **7** analyzed, 3 F; 48±14.5), **Knee OA** (8; **5** analyzed, 1 F; 58.6±4.3) | Lidocaine patch (2 w) | 3T, spontaneous pain rating/painful stimulation (pressure)/visual, MNI, Yes | PRE, POST, PRE vs. POST | Covariate, ROI-based conjunction | Decreased: med. PFC, rACC, sup. frontal, post. Parietal (CLBP) | Decrease: SFMPQ, NPS (CLBP) | - |
|  |  |  |  |  | med. PFC, THAL, INS, SII, mid ACC, mid. frontal | Decreased: mid. frontal, ant. INS, inf. frontal, ACC, THAL (OA) |  |  |
| **4. Geha, 2008 (CT)[19]** | **Postherpetic neuralgia** > 3 mon (14; **11** analyzed, 9 F; 66.4) | Lidocaine patch (2 w) | 3T, painful stimulation (brushing; allodynia)/ light touch/visual, MNI, Yes | PRE + POST | ROI-based correlation | - | Decrease: spontaneous pain | - |
| **5. Harris, 2008 (RCT)[24]** | FM > 1 y (**10**; all F; 48±15) | ACU/sham ACU (9, 4 w) | 3T, painful stimulation (pressure), MNI, mixed | - | ROI-based correlation | - | Decreased: SFMPQ (sensory dimension of pain), pressure-evoked pain sensitivity | - |
|  |  |  |  |  | post. INS |  |  |  |
| ***6. Gustin, 2010 (RCT)[22]** | **Complex regional pain syndrome** > 6 mon (20, 12 F)  -morphine + placebo (10, **7** analyzed; 49.8±10.3)  -morphine + memantine (10, **7** analyzed; 52±13.3) | NMDA-receptor antagonist (memantine, > 5mg, 7 w), morphine (> 10mg, 3 times/d, 8 w) | 3T, hand squeezing/visual, MNI, Yes | PRE, PRE vs. POST | ROI-based correlation | Decreased: ACC (combination), SI (> placebo) | Decreased: spontaneous (both groups), stimulus pain, PDI, CES-D (combination) | M/R |
|  |  |  |  |  | SI, SII,  INS, ACC |  |  |  |
| **7. Grazzi, 2010 (CCT)[21]** | **Migraine with medication overuse** (13, **9** analyzed, all F; 34±9)  **HCs** 11, **9** analyzed, all F; 37±7) | Drug withdrawal (6 mon) | 1.5T, painful stimulation (pressure), MNI, No | PRE, POST | - | - | Decreased: Number of headache d, analgesic use | - |
| **8. Ohn, 2011 (CT)[53]** | **Poststroke central pain** > 6 mon (22, 9 F; **9** analyzed; 54.9±9)  -RES (**5**)  -non-RES (**4**) | Repetitive transcranial magnetic stimulation (1000 pulses/d, 5 d) | 3T, painful stimulation (sand paper scratching), MNI, No | PRE, POST, PRE vs. POST | - | Decreased: SII, INS, PFC, putamen, cerebellum (RES) | Decreased: Pain perception, HDRS | - |
| **9. Parks, 2011 (CCT)[54]** | **Knee OA** > 3 mon (14, 4 F; **6** treated, 0 F; 56.1±2.1)  **HCs** **9**, 3 F; 46.55±2.6) | Cyclooxygenase-2 inhibitor (valdecoxib, 10mg, 3/d, 2 w) | 3T, painful stimulation (pressure), spontaneous pain rating, MNI, Yes | - | Covariate, correlation | - | Decreased: spontaneous pain, SFMPQ, WOMAC (worse knee pain) | - |
| **10. Diers, 2012 (CCT)[12]** | **FM** (**10**, 9 F; 52.1±6.1)  **HCs** (**10**, 7 F; 48.7±11.5) | Extinction training (12, 12 w) | 1.5T, painful stimulation (pressure), pain rating, MNI, Yes | - | ROI-based correlation | - | Decrease:  interference  from pain (MPI)  Increase: pain threshold/tolerance | - |
|  |  |  |  |  | ACC, SI, SII, INS, THAL, PAG, caudate nucleus, lenticular nucleus |  |  |  |
| ***11. Jensen, 2012 (RCT)[29]** | **FM** (43, all F; 45.6±6.4)  -CBT (25, **19** completed; 44.5±1.5)  -WL (18, **15** completed; 46.9±1.1) | CBT/WL (12, weekly) | 1.5T, painful stimulation (pressure). MNI, Yes | PRE, PRE vs. POST | Seed-based PPI, correlation | Increased: inf. frontal, vlPFC, OFC, ant. INS (CBT) | Improved: PGIC  Decrease: BDI, pain | - |
| **12. Hashmi, 2012a (RCT)[27]** | **CLBP** > 1 y (38; **30** analyzed, 14 F; 51.36±9.1)  -Lidocaine 15  -Placebo 15 | Lidocaine patch/placebo (2 w) | 3T, spontaneous pain rating/visual, MNI, Yes | PRE, POST1 (6 h after Tx), POST2 (2 w after Tx) | Correlation | - | Decreased: pain, MPQ scores (sensory, affective; both groups) | - |
| **13. Hashmi, 2012b (RCT**, re-analysis of Hashmi 2012a**)[28]** | **CLBP** > 1 y (**30**, 14 F; 51.36±9)  -RES 15, 7 F  -Non-RES 15, 7 F | Lidocaine patch (2 w) | 3T, spontaneous pain rating, MNI, Yes | PRE, POST | ROI-based FC, spectral power,  whole-brain FC (seed: dlPFC), PCA | - | Pain intensity, MPQ, BDI, anxiety (BAI), NPS | - |
| **14. Ferraro, 2012 (CCT)[14]** | **Medication overuse**  **headache** (13, **9** completed; 34±9)  **HCs** (**9**; 38±7) | Drug withdraw (6 mon) | 1.5T, painful stimulation (pressure), MNI, Yes | PRE, POST, PRE + POST, PRE vs. POST | - | - | Decreased: headache days/severity | - |
| ***15. Chu, 2012 (RCT)[9]** | **IBS** > 3 mon (**30**, 15 F)  -Electro-ACU (**15**, 7 F; 42.3±12.2)  -sham EA (**15**, 8 F; 44.2±14.5) | EA/sham EA | 3T, painful stimulation (rectal distension), EA, TAL, No | PRE, POST, PRE vs. POST | Correlation | Increased: MI, sup. med. frontal, inf. parietal, sup./mid. temporal, fusiform, calcarine, rolandic operculum, sup. occipital, paracentral lobule (EA) | - | - |
| **16. Tillisch, 2012 (RCT**, counterbalanced**)[66]** | **IBS** (16; **8** analyzed, all F; 32.3±12.7) | Neurokinin-1-receptor antagonist (AV608)/placebo (3 w + 2 w washout) | 3T, painful stimulation (rectal distension), MNI, Yes | - | ROI, correlation | - | Decreased: MPQ, anxiety (HADS), negative affect (PANAS) | M/R (sinus congestion, headache, etc.) |
|  |  |  |  |  | AMG, HIPPO, hypoTHAL, (pre/subgneual) ACC, aMCC, ant./post. INS, THAL |  |  |  |
| **17. Taylor, 2013 (RCT)[64]** | **FM** (**46**, 43 F; 50.8±10.4)  -Usual care (15, 14 F; **6** completed; 48.6±9.8)  -Active CES device (17, 16 F; **6** completed; 51.9±10.6)  -Sham device (14, 13 F; **6** completed; 51.5±10.9) | CES/usual care/sham device (8 w, daily) | 3T, painful stimulation (pressure), TAL, Yes | PRE vs. POST | ROI | Decreased: PCC, ACC, THAL | Decreased: SFMPQ, pain (CES) | M |
|  |  |  |  |  |  | Increased: INS, PFC |  |  |
| **18. Lowén, 2013 (CCT)[44]** | **IBS** (44; **31** completed; 35.5)  -Hypnotherapy (25; **16** analyzed)  -EDU (16; **9** analyzed)  **HCs** (20; **18** analyzed; 32.2) | Hypnotherapy/EDU (6, 6 w) | 1.5T, painful stimulation (rectal distention), MNI, Yes | - | ROI, correlation | - | Decreased: IBS-SSS, VSI (both groups) | - |
|  |  |  |  |  | AMG, HIPPO, pre/subgenual ACC, ant. MCC, PAG, THAL, vl/dlPFC, ant./mid./post. INS |  |  |  |
| ***19. Petzke, 2013 (RCT)[55]** | **FM** (**92**, all F)  -Milnacipran (46; **32** analyzed; 45.6±8.5)  -Placebo (46; **32** analyzed; 42.8±7.8) | Milnacipran (200mg/d, 13 w)/placebo | 1.5T, painful stimulation (pressure), MNI, mixed | PRE, PRE vs. POST | ROI | Increased  : SI, ant. INS, ACC, PCC, THAL, AMG, preCUN, caudate nucleus, cerebellum | NS | M/R (nausea, vomiting, etc.) |
|  |  |  |  |  | rACC, brain stem, AMG, caudate nucleus, ant./post. INS, SI, SII, ACC |  |  |  |
| ***20. Kim, 2013 (CCT)[31]** | **FM** (**21**, all F; 51.3±8.4)  -Pregabalin RES (9; **7** analyzed)  -Non-RES (12)  **HCs** (**11**, all F; 46.5±12) | Pregabalin | 3T, painful stimulation (pressure), MNI, mixed | PRE, POST, PRE vs. POST | - | Decreased: THAL, SI,  IPL, calcarine, mid.  frontal, MCC, preCUN, INS | Decreased: pressure pain sensitivity, FIQ, BFI, BDI, WPI, SSS | - |
| **21. Foell, 2013 (CT)[16]** | **Phantom limb pain** > 2 y (13, 4 F; analyzed 11, 4 F; 50.6±15.8) | Mirror therapy (4 w, daily) | 3T, mirrored hand movement/lip pursing, MNI, mixed | PRE, POST, PRE vs. POST | ROI-based correlation, regression, cortical lip representation | NS | Decreased: WHYMPI | - |
|  |  |  |  |  | SI, SII, MI, ACC, INS, parietal |  |  |  |
| **22. Jensen, 2014** (**RCT**, re-analysis of Petzke, 2013**)[30]** | **FM** (**92**, all F; 44±8.2)  -Milnacipran (46; **30** analyzed; **21** RES)  -Placebo (46; **30** analyzed; **16** RES) | Milnacipran (200mg/d, 12 w)/placebo | 1.5T, painful stimulation (pressure), MNI, mixed | PRE, POST | ROI, correlation, conjunction | - | Decreased: FIQ (RES) | M |
|  |  |  |  |  | PCC |  | Increased: P50 (stimulus-response assessments, RES) |  |
| **23. Zhu, 2014 (RCT)[74]** | **D-IBS** (**80**)  -Mox (40; **15** analyzed, 6 F; 47.5±0.9)  -Sham Mox (40; **13** analyzed, 6 F; 40.9±10.1) | Mox (3 times a week, 4 w) | 1.5T, painful stimulation (rectal distention), MNI, Yes | PRE, POST | - | - | Decreased: pain, B-IBS-SS, IBS-QOL | - |
|  |  |  |  |  |  |  | Increased: pain/defecation urge threshold |  |
| **24. Zhao, 2015 (RCT)[72]** | **D-IBS** (62)  -Mox (30; **8** analyzed; 39.5±8.9)  -EA (32; **7** analyzed; 42.8±10.2)  **HCs** (**7**) | Mox/EA | -, painful stimulation (colorectal distension), -, - | PRE, POST1 (1 mon after Tx), POST2 (3 mon after Tx) | - | - | Decreased: abdominal pain/distension, defecation emergency/frequency, HARS/HDRS, rectal sensory ratings | M/R |
|  |  |  |  |  |  |  | Increased: pain/defecation perception threshold |  |
| ***25. Sanders, 2015 (RCT**, cross-over**)[59]** | **Hand OA** > 6 mon (23; **19** analyzed, 18 F; 60.7±6.4) | Naproxen (500mg, 2/d, 1 w)/placebo | 3T, hand squeezing, MNI, No | POST vs. PRE | ROI, correlation | Decreased: THAL, SI, INS, MI, lentiform nucleus, caudate, putamen, claustrum, PCC, IPL, (pre)CUN, supramarginal, lingual, mid. occipital, paraHIPPO, fusiform, transverse temporal, AMG, ant./post. cerebellar lobe, vermis, uvula | Decreased: pain, MPQ (sensory), wrist/hand pain and function (WHE) | M |
|  |  |  |  |  | THAL, SI, SII, HIPPO  formation, ant./post. INS, ACC, PCC, AMG |  |  |  |
| ***26. Geha, 2016 (CCT**, cross-over**)[17]** | Inherited erythromelalgia (**2**, 1 F) | Carbamazepine (> 200mg/d, 4 w)/placebo | 3T, painful stimulation (thermal heat), spontaneous pain rating/visual, MNI, Yes | POST vs. PRE | Correlation | Decreased: SI, MI, parietal | Decreased: Mean pain time/duration | M |
|  |  |  |  |  |  | Increased: rACC, PCC, NAc |  |  |
| **27. Zhao, 2018 (RCT)[73]** | **C-IBS** (63)  -EA (31, **7** completed; 40.4±12.7)  -Mox (32, **6** completed; 42.3±8.7)  **HCs** (**7**) | Mox/EA (30 min, 6 times/w, 4 w) | 1.5T, painful stimulation (rectal distension), -, No | PRE, PRE vs. POST | - | Decreased: ACC, INS, PFC (EA) | Decreased: IBS symptoms (both groups), defecation frequency, difficulty in defecation, HARS/HDRS pain intensity (EA) | M/R |
|  |  |  |  |  |  |  | Increased: stool form (BSFS), defecation/pain threshold (EA) |  |
| **Non-pain-related task studies** | | | | | | | | |
| **28. Napadow, 2007 (CCT)[50]** | **Carpal tunnel syndrome** > 3 mon (13; **10** analyzed, 6 F; 51.1)  **HCs** (12; **9** analyzed; 46.9) | ACU, sham ACU (13, 5 w) | 3T, ACU, MNI, Yes | PRE, PRE vs.  POST | FC | Decrease: lateral hypoTHAL  Increase: vmed. PFC, SI, ant./post. INS, ACC, AMG | - | - |
| **29. Seminowicz, 2011 (CCT)[61]** | **CLBP** > 1 y (18, 10 F; analyzed **14;** 46±10.6)  -spine surgery (**8**)  -zygapophysial joint block (**6**)  **HCs** (16, 8 F; analyzed 10, 6 F; 40±13.2) | Spine surgery/zygapophysial joint injections | 3T, Multi-Source Interference Task, MNI, Yes | PRE, POST, PRE vs. POST | Correlation | Decreased: dlPFC | Decrease: SFMPQ, ODI, depression (Profile of Mood States, RES) | - |
| **30. Micalos, 2014 (CCT)[49]** | **FM** > 1 y (8), back pain (2), complex regional pain (1; 50±12)  **HCs** (**8**, 7 F; 49.6±10) | Aerobic exercise (2/w, 12 w) | 3T, non-painful stimulation (pressure), MNI, Yes | PRE | ROI | - | NS | - |
| **31. Harte, 2016 (RCT**, cross-over, re-analysis of Harris, 2013**)[26]** | **FM** > 6 mon (42, **17** treated, all F; 40.9±10.7)  **HCs** (20, **18** analyzed, all F; 41.4±11.9) | Pregabalin (450mg/d, 17 d)/placebo | 3T, visual, MNI, Yes | PRE, PRE vs. POST | Correlation, MVPA | Decreased: ant. INS | - | - |
| **32. Braden, 2016 (CCT)[4]** | **CLBP** (26, 17 F)  -MBSR (13, **12** analyzed, 8 F; 46±11.3)  -reading control (13, **11** analyzed, 6 F; 43±2.5)  **HCs** (11) | MBSR course (4 w) | 3T, sadness induction/visual, MNI, No | - | ROI-based correlation | - | Decreased: BDI-II (both), Oswestry Low Back Pain Scale (MBSR) | - |
|  |  |  |  |  | dm/vlPFC, ant. INS, subgenual ACC |  |  |  |
| **33. Pinto-Sanchez, 2017 (RCT)[56]** | **IBS** (44, 24 F)  -Probiotic (22, 12 F; **18** completed; 46.5)  -Placebo (22, 12 F; **20** completed; 40) | Bifidobacterium longum NCC3001/placebo (daily, 6 w) | 3T, visual emotional face, TAL, Yes | PRE, POST, PRE + POST | ROI-based  correlation | - | Decreased: HADS  Improved: quality of life (short-form healthy survey) | M/R |
|  |  |  |  |  | AMG |  |  |  |
| **34. Maeda, 2017 (RCT)[46]** | **Carpal tunnel syndrome** > 3 mon (79, 64 F; 49.3±8.6)  -local EA (28, 22 F; 11 analyzed; 48.5±10.1)  -distal EA (28, 22 F; **13** analyzed; 49.9±8.4)  -sham EA (23, 20 F; **12** analyzed; 50.6±7.8) | EA/sham EA (16, 8 w) | 3T, vibrotactile stimulation, MNI, Yes | - | ROI-based digit separation distance, correlation | - | Decreased: BCTQ, symptom severity (all groups after Tx, verum EA after 3 mon follow-up), median sensory nerve latency (verum ACU) | - |
|  |  |  |  |  | SI |  |  |  |
| **35. Privitera, 2017 (CT)[57]** | Limb amputation (14, 8 F; **9** analyzed; 53) | Capsaicin 8% patch (3-4 w) | 3T, lip pursing, MNI, Yes | PRE + POST | ROI | - | Decreased: spontaneous/stimulus pain, SFMPQ, area of allodynia/hypersensitivity  Improved: PGIC | M/R |
|  |  |  |  |  | MI |  | Increased: thermal threshold |  |
| **36. Gray, 2018 (CT)[20]** | **Crohn’s disease** > 6 mon (11; **9** analyzed, 5 F; 26.1±10.6)  -adalimumab (**5**; 29.2±13.7)  -infliximab (**4**; 22.3±4.1) | Anti-tumor necrosis factor α (various) | 3T, implicit associations test, MNI, Yes | Interaction of Tx and classification condition | ROI, correlation | Interaction in the post. inf. temporal | Decreased: overall symptom response | - |
|  |  |  |  |  | PFC, cingulate, AMG |  |  |  |
| **37. Timmers, 2019 (CCT)[67]** | **CLBP** > 6 mon (23; **14** analyzed, 3 F; 42.4±11.6)  -POST1 (10, 1 F)  -POST2 (9)  **HCs** (14, 4 F; **10** analyzed, 2 F; 41.7±12.5) | Exposure in vivo (various) | 3T, MNI, visual imagination, Yes | PRE, POST,  PRE vs. POST1, POST2 (6 mon follow-up) | ROI, correlation | Decreased: SI, MI, supramarginal (PRE vs. POST1), PFC, mid. frontal, SI, angular/inf. parietal lobe, (PRE vs. POST2) | Decreased: Pain-related fear/disability, perceived activity decline, performance tasks |  |
|  |  |  |  |  | med. frontal, AMG, NAc, HIPPO | Increased: preCUN (PRE vs. POST1) |  |  |

**Supplementary table 2. Resting fMRI studies**

| **No. Author, year (study type)** | **Patients, disease duration, no.** | **Treatment (no., duration)** | **Imaging, whole-brain voxel-wise analysis, standard template, correction** | **Other analyses** | **PRE vs. POST of brain/clinical/behavioral outcomes** | **Adverse events** |
| --- | --- | --- | --- | --- | --- | --- |
|  |  |  |  | **ROIs** |  |  |
| **1. Napadow, 2012 (CT)[51]** | **FM** > 1 y (**17**, all F; 46.4±15.5) | ACU/sham ACU (9, 4 w) | 3T, REST, MNI, Yes | ICA (DMN), FC, regression (DMN connectivity-pain) | Decreased: SFMPQ (sensory subscore), connectivity between the DMN-ant./mid. INS, DMN-putamen. | - |
| **2. Li, 2014 (CCT)[38]** | **CLBP** > 3 mon (20, 10 F; **18** analyzed; 38.1±6.4)  **HCs** (**10**, 5 F; 37.7±5.1) | ACU (12, 4 w) | 3T, REST, MNI, Yes | ICA (DMN), correlation (DMN connectivity-pain) | Decreased: pain | - |
|  |  |  |  |  | Increased: DMN voxel no., DMN connectivities, connectivities of the dlPFC, med. PFC, ACC, preCUN |  |
| **3. Chen, 2014 (RCT)[8]** | **Knee OA** > 3 mon (44; 30 analyzed, 13 F; 58±8)  -High ACU (**10**, 2 F; 60±9)  -Low ACU (**10**, 7 F; 58±8)  -Sham ACU (**10**, 4 F; 54±7) | High ACU/low ACU/sham ACU (6, 1 mon) | 3T, REST + task (ACU), TAL, Yes | FC (seed: post. med. PFC) | Improved: KOOS (pain, other symptoms, function in sport, quality of life) | - |
|  |  |  |  |  | Increased: FC of post. med. PFC-ant. med. PFC, rACC, PAG, ventral striatum |  |
| **4. Shpaner, 2014 (RCT)[62]** | **Musculoskeletal pain** > 1 y (38, **28** analyzed)  -CBT (19, 16 F; 43.6 ±13.7)  -EDU (19, 13 F; 39.2±14.1) | CBT (11, weekly) | 3T, REST, MNI, mixed | ICA (DMN, salience network, basal ganglia network), ROI-based FC, fALFF, correlation | Decreased: pain symptoms, Passive Coping (TOPS), connectivity of DMN-AMG, PAG, occipital (> EDU) | - |
|  |  |  |  |  | Increased: Mental Composite Score, Self-Efficacy for Pain Management, Self-Efficacy for Coping with Symptoms, FC of BG-SII, fALFF in cerebellum, PCC, FC in cerebellum, PCC, preCUN, THAL (> EDU) |  |
| **5. Egorova, 2015 (RCT**, sample overlaps with Chen 2014**)[13]** | **Knee OA** > 3 mon (44; 30 analyzed, 13 F; 57.5±8.3)  -High ACU (**10**, 2 F)  -Low ACU (**10**, 7 F)  -Sham ACU (**10**, 4 F) | High ACU/low ACU/sham ACU (6, 1 mon) | 3T, REST + task (ACU), MNI, Yes | FC (seed: PAG), correlation | Decrease: KOOS pain, function in sport, correlation between FC PAG-HIPPO and pain | - |
| **6. Flodin, 2015 (CCT)[15]** | **FM** (16; **14** analyzed, all F; 48.4)  **HCs** (**11**, all F; 41.8) | Physical exercise (30, 15 w) | 3T, REST, MNI, Yes | FC (seed: INS, supramarginal, MCC, THAL), correlation | Decreased: FIQ, FC supramarginal -cerebellum | M |
|  |  |  |  |  | Increased: FC ant. INS-primary sensory-motor area |  |
| **7. Li, 2015 (CCT)[39]** | **Migraine** > 1 y (**12**, 10 F; 28.1±6.8)  **HCs** (**12**, 10 F; 29.8±7.2) | ACU (5/w, 4 w) | 3T, MNI, Yes | ICA-based FC (frontoparietal network), correlation | Decreased: pain, duration and frequency of migraine attacks | M/R |
|  |  |  |  |  | Increased : FC with the FPN in  the MI, IPL, SI |  |
| **8. Cummiford, 2016 (CT**, cross-over**)[10]** | **FM** > 1 y (13; analyzed **12**, all F; 47.6±10.6) | tDCS, sham tDCS (5 d) | 3T, REST, MNI, Yes | FC (seed: MI, SI, THAL, PAG), regression | Decreased: pain, PANAS negative affect, FC VL THAL-IPL, PAG-PCC | - |
| **9. Zhang, 2016 (CCT**, sample overlaps with Li, 2015**)[71]** | **Migraine** > 1 y (**12**, 10 F; 28.1±6.8)  **HCs** (**12**, 10 F; 29.8±7.2) | ACU (5/w, 4 w) | 3T, MNI, Yes | ICA-based FC | Decreased: pain, PSQI, duration and frequency of migraine attacks | M/R |
|  |  |  |  |  | Increased: FC in med./sup. frontal, inf. parietal lobule, PCC, cingulate, mid./sup. temporal, supramarginal |  |
| **10. Li, 2016 (RCT)[40]** | **MwoA** > 6 mon (100; **62** analyzed; 21.3)  -ACU (**35**, 27 F)  -sham ACU (**11**, 9 F; 21.2)  -WL (**16**, 12 F; 21.4)  **HCs** (46; **42** analyzed, 34 F; 21.2) | ACU (20, 4 w) | 3T, REST, Yes, MNI, Yes | FC (seed: vlPAG), regression (FC-migraine symptom) | Decreased: Headache intensity, frequency | - |
|  |  |  |  |  | Increased: FC vlPAG-, rACC, med. PFC, MCC |  |
| **11. Yoshino, 2017 (CCT)[70]** | **Somatoform pain disorder** (**29**, 20 F; 46.7±11.3)  **HCs** (**30**, 25 F; 46.9±10.3) | CBT (12, weekly) | 3T, REST, MNI, Yes | ROI-based ICA (DMN, sensorimotor network, central executive network, dorsal attention network), correlation | Decreased: Pain, SFMPQ, PCS, BDI-II, STAI, ICN connectivity in IPL, paracentral lobule | - |
|  |  |  |  | med. PFC, inf. frontal, OFC, sup./mid. temporal, IPL, PCC, THAL, lingual, putamen, paracentral lobule | Increased: OFC ICN connectivity |  |
| **12.** [**Lazaridou, 2017**](https://www.ncbi.nlm.nih.gov/pubmed/?term=Lazaridou%20A%5BAuthor%5D&cauthor=true&cauthor_uid=27518491) **(RCT)[37]** | **FM** > 1 y (16; 45.7±12.2)  -CBT (**8**)  -FM EDU (**8**) | CBT/EDU (4, 4 w) | 3T, REST, MNI, Yes | FC (seed: SI), regression | Decreased: PCS, pain, FC of SI-ant./med. INS (CBT > EDU), pain interference (BPI) | - |
| **13. Li, 2017 (RCT**, sample overlaps with Li, 2016**)[41]** | **MwoA** > 6 mon (100; **62** analyzed; 21.3)  -ACU (**35**, 27 F)  -sham ACU (**11**, 9 F; 21.2)  -WL (**16**, 12 F; 21.4)  **HCs** (46; **42** analyzed, 34 F; 21.2) | ACU (20, 4 w) | 3T, REST, Yes, MNI, Yes | ALFF, regression | Decreased: Headache intensity/frequency, ALFF in mid. occipital, CUN | - |
|  |  |  |  |  | Increased: ALFF in medulla, trigeminocervical complex (> sham ACU, WL), OFC (> WL), midbrain |  |
| **14. Krebs, 2018 (CCT)[35]** | **Migraine with medication overuse** (14; **10** analyzed, all F; 43.3±13.1)  **HCs** (**10**, all F; 41.9±11.5) | Sphenopalatine ganglion blockade (12, 6 w) | 3T, REST, MNI, Yes | ROI-based FC | Decreased: headache days/impact test score, patient health questionnaire (PHQ-9), medication use, FC sup. temporal-SMG | - |
|  |  |  |  | OFC, INS, dACC, (ant./dorsal/dl/vl/dm) PFC, inf. frontal, frontal eye fields, frontal operculum, hypoTHAL, paracingulate, AMG, supramarginal, PAG, (pre)SMA, ventral tegmental area/substantia nigra, (sup./inf.) temporal pole, ventral striatum/pallidum, ant. THAL, lateral parietal, dorsal/vm caudate | Improved: FC of ant. PFC-OFI, vSP, SMA, dlPFC, VTA/SNPC-dlPFC, temporal pole, dlPFC-ant. THAL, vm Caudate, dPFC/FEF, vlPFC, dPFC/FEF-THAL, vm caudate, dPFC/FEF-Caudate, vlPFC |  |
| **15. Kong, 2018 (RCT)[34]** | **Knee OA** > 3 mon (74; **46** analyzed, 27 F)  -Boosted ACU (**17**, 9 F; 61.3±6.9)  -Standard ACU (**17**, 10 F; 61.2±7.7)  -Usual Tx (**12**, 8 F; 60.1±7.1) | Boosted ACU/standard ACU/Tx as usual (6, 4 w) | 3T, REST + heat pain, MNI, Yes | FC (seed: NAc), regression | Decreased: KOOS pain (boosted > standard, TAU)  Increased: FC of NAc-med. PFC, rACC, dlPFC, MCC, paracentral, SI (boosted > standard), NAc-vmed. PFC (boosted > TAU), expectancy-PRE vs. POST FC of NAc-rACC, med. PFC | - |
| **16. Chen, 2018 (CCT)[6]** | **Cervical spondylosis neck pain** > 3 mon (**104**, 59 F; 24.9±2)  **HCs** (**96**, 46 F; 24.8±1.5) | ACU (10, 4 w) | 3T, REST, MNI, Yes | ReHo, correlation, MVPA | Decreased: NPQ | - |
|  |  |  |  |  | Increased: ReHo in TPJ |  |
| **17. Zou, 2019 (CCT)[75]** | **Migraine without aura** > 3 mon (35; **14** analyzed, 9 F; 42.7±10.2)  **HCs** (25; **18** analyzed, 9 F; 38.6±8) | ACU (36, 3 mon) | 3T, REST, MNI, Yes | ICA (DMN), ROI-based FC, correlation | Decreased: pain, headache attacks/ days, medication use | - |
|  |  |  |  | ACC, preCUN, sup. med., sup. prefrontal, temporal lobe | Increased: DMN connectivities, FC of TPL-ACC, TPL-SMG, TPL-preCUN |  |
| **18. Tétreault, 2019 (RCT)[65]** | **Knee OA** > 1 y (70; **39** analyzed, 22 F; 58.7±7.6)  -Duloxetine (**19**)  -Placebo (**20**)  **HCs** (**20**, 10 F; 57.9±6.7) | Duloxetine/placebo (30/60mg, daily, 13 w) | 3T, REST, MNI, Yes | Graph construction, regression, mediation | Decreased: WOMAC (RES) | M/R |
|  |  |  |  |  | Changed: degree count of frontal pole, inf. temporal, preCUN, sup. parietal lobule |  |
| **19. Neeb, 2019 (RCT)[52]** | **Inflammatory bowel disease** > 3 mon (36, F 28; **31** analyzed; 35.4±12.9)  -Active tDCS (Crohn’s disease + ulcerative colitis; 24, 18 F; **21** analyzed)  -Sham tDCS (Crohn’s disease; 12, 10 F; **10** analyzed) | tDCS on MI (5 d) | 3T, REST, MNI, Yes | FC | Decreased: LFO in PCC, preCUN, FC of DMN-sensorimotor network (SI, MI), salience network (MCC, TPJ) (RES) | - |
|  |  |  |  |  | Increased: FC within visual med. network, INS, SI, MI, cingulate, temporal, frontal, FC of fronto-parietal network-cingulate, callosal body |  |
| **20. Rogachov, 2019 (CCT)[58]** | **Neuropathic pain** > 4 mon (30, F 14; 42.8±14.4)  -RES (**15**, 8 F)  -non-RES (**15**, 6 F)  **HCs** (**33**, 17 F; 42.5±12.6) | Ketamine (0.5-2.0 mg/kg/h, 6 h/d, 5 d) | 3T, REST, MNI, Yes | Low-frequency brain oscillations (LFO) in ROIs, ROI-based FC, machine learning | Decreased: pain (BPI), % pain relief (RES > non-RES) | M |
|  |  |  |  | THAL, SI, DMN (PCC, med. PFC) |  |  |
| **21. De Groote, 2020 (CT)[11]** | **Failed back surgery syndrome** > 1 y (**10**, F 8; 54.7±6.3) | 10kHz high frequency spinal cord stimulation (3 mon) | 3T, REST, MNI, Yes | ROI-based FC, correlation | Increased: FC of dlPFC-ant. INS, sensorimotor, ant. INS-inf. parietal, ACC-cerebellar post. network | - |
|  |  |  |  | DMN, sensorimotor network, salience network, frontoparietal network, central executive network, cerebellar network | Decreased: PCS, PSQI, pain, FC of paracentral lobule-lateral PFC |  |

**Supplementary table 3. Resting + task fMRI studies**

| **No. Author, year (study type)** | **Patients, disease duration, no.** | **Treatment** | **MRI, stimulation and task, standard template, correction** | **Analyses** | **PRE vs. POST in the brain** | **PRE vs. POST of clinical/behavioral outcomes** | **Adverse events** |
| --- | --- | --- | --- | --- | --- | --- | --- |
|  |  |  |  | **ROIs** |  |  |  |
| **1. Harris, 2013 (RCT**, cross-over**)[23]** | **FM** > 6 mon (27 all F; **14** analyzed; 37.8±11) | Pregabalin/placebo (450 mg/d, 2 w) | 3T, REST + painful stimulation (pressure), MNI, Yes | Seed-based FC (seed: INS), regression, correlation, task related GLM | Decreased: DMN (IPL, PCC) (pain; > placebo) | Decreased: spontaneous pain (pregabalin) | M |
| **2. Chen, 2015 (RCT**, sample overlaps with Chen 2014**)[7]** | **Knee OA** > 3 mon (44; 30 analyzed, 13 F; 58±8)  -High ACU (**10**, 2 F; 60±9)  -Low ACU (**10**, 7 F; 58±8)  -Sham ACU (**10**, 4 F; 54±7) | High ACU/low ACU/sham ACU (6, 1 mon) | 3T, REST + ACU, MNI, Yes | ICA (REST), regression, task related GLM | Decreased: FC of somatosensory network-dorsal ACC, frontal (> sham) | Decreased: KOOS pain score (> sham) | - |
|  |  |  |  |  | Increased:  post. operculum/SII, CUN  FC of frontoparietal network, executive control network-rACC, med. PFC, INS  Correlation between rFPN-INS FC and KOOS pain score (> sham) |  |  |
| **3. Ceko, 2015 (CCT)[5]** | **CLBP** > 1 y (**14**)  **HCs** (16; **10** analyzed) | Spine surgery (8)/facet joint block (6) | 3T, REST + multisource interference, MNI, Yes | Task-related ICA, FC, ROI-based FC, correlation | Decrease: FC of dlPFC-sACC, vmed. PFC | Decreased: ODI | - |
|  |  |  |  | INS, dlPFC | Increased: FC of task-related network-INS, task-positive network-dlPFC, ant./mid. INS-dlPFC, vlPFC, SMA, med. PFC, PCC, preCUN, temporal, visual, ant. INS, frontal operculum, dlPFC-pMCC, SI, MI, PMC, PPC, cerebellum, fusiform, visual, temporal |  |  |
| **4. Smallwood, 2016 (CT)[63]** | **CLBP** > 1 y (25)  -Chronic pain focused ACT (**6** completed, 2 F; 43.5±11.5)  -Health EDU (**6** completed, 3 F; 49.7±7.1) | Acceptance + commitment therapy/EDU (8, 4 w) | -, REST + painful stimulation (pressure), MNI, No | Seed-based FC (seed: DMN and pain regions; INS, PCC, SI, SII, AMG, THAL, ACC, OFC, sup. frontal, inf. temporal, paraHIPPO, lateral parietal), correlation | Increased: FC of post. INS-sup. PCC, inf. temporal -inf. PCC | - |  |
|  |  |  |  |  | Decreased: mid. frontal, inf. parietal lobule, INS, ACC, PCC, sup. temporal (ACT), FCs in DMN/pain regions |  |  |

**Supplementary table 4. PET studies**

| **No. Author, year (study type)** | **Patients, disease duration, no.** | **Treatment (no. duration)** | **PET imaging, stimulation and task, standard template, correction** | **Analyses** | **PRE vs. POST in the brain** | **PRE vs. POST of clinical/behavioral outcomes** | **Adverse events** |
| --- | --- | --- | --- | --- | --- | --- | --- |
| **Glucose PET studies** | | | | | | | |
| **1. Walitt, 2007 (CT)[68]** | **FM** (12; **9** analyzed, 9 F; 47.7) | Individualized, comprehensive Tx (8 w) | 18F-FDG, REST, TAL and Tournoux, Yes | Volumes of interest, linear relationship | Increased: OFC, med/sup frontal, INS, THAL, putamen, caudate nucleus, brain stem, cingulate, rectus, (para)HIPPO, preCUN, paracentral lobule | Decreased: FIQ, tender point | M/R |
| **2. Magis, 2011 (CCT)[47]** | **Drug-resistant chronic cluster headache** > 2 y **(10**, 1 F; 44.2±9.9)  **HCs** (**39**, 21 F; 45±16) | Occipital nerve stimulation (various) | 18F-FDG, occipital nerve stimulation, TAL, Yes | Whole-brain, ROI | Decreased: ACC, MCC, pulvinar, midbrain, pons, visual, cerebellum | 7 responders, 3 non-responders (pain, number/frequency of attacks) | - |
|  |  |  |  |  | Increased: sensorimotor |  |  |
| **3. Yoon, 2014 (CCT)[69]** | **Neuropathic pain** > 3 mon (**16**, F 4; 44.1±8.6) | tDCS on MI/sham tDCS (20, 10 d) | 18F-FDG, REST, MNI, No | Whole-brain, correlation, regression | Decreased: PCC, OFC, dlPFC, frontal (> sham), angular, preCUN (tDCS) | Decreased: pain, pain interference (tDCS) | - |
|  |  |  |  |  | Increased: SI, INS, subgenual ACC, caudate (> sham), fusiform, (para)HIPPO, putamen, brainstem, medulla (tDCS) |  |  |
| **4. Boyer, 2014 (RCT)[3]** | **FM** > 6 mon (38)  -TMS (**19**, F 19; 49.1±10.6)  -sham (**19**, F 18; 47.7±10.4) | High frequency TMS on MI/sham (14, 10 w) | 18F-FDG, REST, -, No | Whole-brain, correlation | Increased: (para)HIPPO, fusiform, Brodmann area 20 (> sham) | Decreased: FIQ (> sham) | M/R |
|  |  |  |  |  |  | Increased: mental composite score of short form-36 (> sham) |  |
| **5. Sawaddiruk, 2019 (RCT**, cross-over**)[60]** | **FM** (**11**, F 9; 46±11) | Pregabalin + CoQ10/pregabalin + placebo (40 d) | 18F-FDG, REST, TAL, Yes | Whole-brain | Decreased: temporal lobe, cerebellum (> baseline, placebo at day 80 only) | Decreased: pain, Thai-HDRS, FIQ (> placebo) | - |
|  |  |  |  |  |  | Increased: pain pressure threshold (> placebo) |  |
| **Blood flow PET studies** | | | | | | | |
| **6. Berman, 2002 (RCT)[2]** | **non-C IBS** (52; **37** completed; 40.7)  -Alosetron (**20**, F 9; 39.2**)**  -placebo (**17**, F 9; 40.7) | Alosetron/placebo (twice daily, 3 w) | H_2_^15^O, visceral distention + sham distention, MNI, Yes | Whole-brain, ROI, covariate | Decreased: ventral striatum, hypoTHAL, infragenual ACC (baseline), AMG (baseline distention, anticipation)  Increased: ant INS (distention, anticipation) | Decreased: pain intensity/unpleasantness, distention unpleasantness (> placebo) | - |
| **7. Mayer, 2002** **(RCT**, sample overlaps with Berman, 2002**)[48]** | **non-C IBS** (52; **37** completed; 40.7)  -Alosetron (**20**, F 9; 39.2**)**  -placebo (**17**, F 9; 40.7) | Alosetron/placebo (twice daily, 3 w) | H_2_^15^O, visceral distention + sham distention, MNI, No | Whole-brain, ROI | Decreased: AMG, ventral striatum, hypoTHAL, infragenual ACC (> placebo), OFC, rACC, temporal, paraHIPPO | Decreased: pain intensity/ unpleasantness, anger to distention | - |
|  |  |  |  |  | Increased: dlPFC, INS, occipital, temporal |  |  |
| **8. Lieberman, 2004 (RCT**, sample overlaps with Berman, 2002**)[42]** | **non-C IBS** (23; **14** analyzed, F 8; 40.7) | Placebo regimen (3 w) | ^15^O-water, visceral distention, TAL, No | Whole-brain, ROI-based FC, regression | NS | Symptom improved (symptom diary) | - |
| **9. Lackner, 2006 (CCT)[36]** | **IBS** > 3 mon (8; **6** analyzed, F 6; 33±9.4)  **HCs** (**5**, F 5; 33±9.8**)** | Cognitive therapy (10, weekly) | ^15^O-water, REST + rectal distention, TAL, Yes | Whole-brain, ROI, covariance | Decreased: ACC, PCC, paraHIPPO, mid/sup temporal, inf/sup frontal | Decreased: IBS symptoms, anxiety, defecation distress, pain severity (short form-36), distention induced unpleasantness, defecation distress, urge, anxiety | - |
| **10. Kishima, 2010 (CT)[32]** | **Neuropathic pain >** 31 mon (**9**, F 3; 47.8±11.9) | Spinal cord stimulation (> 6 mon) | ^15^O-water, REST, TAL, Yes | Whole-brain, correlation, covariance | Increased: THAL, inf/sup parietal, ACC, dlPFC, OFC, MI | Decreased: Pain (SFMPQ) | - |
| **Other PET studies** | | | | | | | |
| **11. Maarrawi, 2007 (CT)[45]** | **Neuropathic pain** > 2 y (**8**, F 3; 54.9±10.7) | MI stimulation (7 mon) | ^11^C-diprenorphine (opioid), REST, International Consortium for Brain Mapping (ICBM), Yes | Whole-brain, ROI, correlation | Decreased: PFC, MCC, PAG, cerebellum | Decreased: Pain | - |
| **12. Harris, 2009 (RCT)[25]** | **FM** > 1 y (**20**, F 20; 44.3±13.6)  -ACU (**10**)  -sham (**10**) | ACU/sham (8, 4 w) | ^11^C-carfentanil PET (μ-opioid), ACU, MNI, Yes | Whole-brain, ROI, correlation | Increased: ACC, putamen, INS, caudate, THAL, AMG, NAc (> sham) | Decreased: Pain (SFMPQ) | - |
| **13. Linnman, 2016 (RCT)[43]** | **Chronic tennis elbow** > 3 mon (10, F 5; **8** completed; 48.7±8.5)  **HCs** (**18**, F 9; 35±9) | Exercise (daily, 3 mon) | ^11^C-GR205171 (Neurokinin-1), REST, MNI, Yes | Whole-brain, ROI | NS | Decreased: Pain, disability | - |

**Abbreviations**

(r)ACC: (rostral) anterior cingulate cortex; ACU: acupuncture; ACT; AMG: amygdala; ant.: anterior; BAI: Beck Anxiety Inventory; B-IBS-SS: Birmingham Irritable Bowel Syndrome Symptom Scale; BCTQ: Boston Carpal Tunnel Syndrome Questionnaire; BDI: Beck Depression Inventory; BFI: Brief Fatigue Inventory; BG: basal ganglia; BPI: Brief Pain Inventory; BSFS: Bristol Stool Form Scale; CBT: cognitive behavioral therapy; CCT: clinical controlled trial; CES: cranial electrical stimulation; CES-D: Center for Epidemiologic Studies Depression Scale; CGIC: Clinical Global Impression of Change; (c-)IBS: irritable bowel syndrome (with constipation); CLBP: chronic low back pain; CT: clinical trial; CUN: cuneus; d: day(s); (d-)IBS: irritable bowel syndrome (with diarrhea); (d)l: (dorso)lateral; DMN: default mode network; EA: electro-acupuncture; EDU: education; F: female; (f)ALFF: (fractional) amplitude of low frequency fluctuations; FC: functional connectivity; FDG: fluorodeoxyglucose; FIQ: Fibromyalgia Impact Questionnaire; FM: fibromyalgia; FPN: fronto-parietal network; h: hour(s); GLM: general linear model; HADS: Hospital anxiety and depression scale; HARS: Hamilton Anxiety Rating Scale; HCs: healthy controls; HDRS: Hamilton Depression Rating Scale; HIPPO: hippocampus; SSS: Severity Scoring System; QOL: Quality of Life; ICA: independent component analysis; inf.: inferior; INS: insula; IPL: inferior parietal lobule; mon: month(s); KOOS: knee injury and osteoarthritis outcome score; MBSR: mindfulness-based stress reduction; MCC: mid-cingulate cortex; med.: medial; MI: primary motor cortex; mid.: middle; MNI: Montreal Neurological Institute; Mox: moxibustion; MPI: Multidimensional Pain Inventory; M/R: monitored and reported; MRI: magnetic resonance imaging; MVPA: multi-voxel pattern analysis; NAc: nucleus accumbens; No: number; non-C: non-constipated; NPS: Neuropathic Pain Scale; NS: not significant; OA: osteoarthritis; ODI: Oswestry Disability Index; OFC: orbitofrontal cortex; PAG: periaqueductal gray; PANAS: Positive and Negative Affect Schedule; PCA: principal component analysis; PCC: posterior cingulate cortex; PCS: Pain Catastrophizing Scale; PDI: Pain Disability Index; PFC: prefrontal cortex; PGIC: Patient Global Impression of Change; post.: posterior; POST: post-treatment; PPI: psychophysiological interaction; PRE: pre-treatment; PSQI: Pittsburgh sleep quality index; RCT: randomized controlled trial; RES: responder; REST: resting-state; ROI: region of interest; (SF)MPQ: (Short-form) McGill Pain Questionnaire; SD: standard deviation; SI: primary somatosensory cortex; SII: secondary somatosensory cortex; SMA: supplementary motor area; SSS: Symptom Severity Scale; sup.: superior; T: tesla; TAL: Talairach coordinates; tDCS: transcranial direct current stimulation; THAL: thalamus; TMS: transcranial magnetic stimulation; Tx: treatment; (v)l: (ventro)lateral; (v)m: (ventro)medial; VSI: visceral sensitivity index; w: week(s); WHE: Wrist and Hand Evaluation; WHYMPI: West Haven-Yale Multidimensional Pain Inventory; WL: waiting list; WOMAC: Western Ontario and McMaster Osteoarthritis Index; WPI: Widespread Pain Index; y: year(s)

**References**

[1] Baliki MN, Geha PY, Jabakhanji R, Harden N, Schnitzer TJ, Apkarian AV. A preliminary fMRI study of analgesic treatment in chronic back pain and knee osteoarthritis. Mol Pain 2008;4:47.

[2] Berman SM, Chang L, Suyenobu B, Derbyshire SW, Stains J, Fitzgerald L, Mandelkern M, Hamm L, Vogt B, Naliboff BD, Mayer EA. Condition-specific deactivation of brain regions by 5-HT3 receptor antagonist Alosetron. Gastroenterology 2002;123(4):969-977.

[3] Boyer L, Dousset A, Roussel P, Dossetto N, Cammilleri S, Piano V, Khalfa S, Mundler O, Donnet A, Guedj E. rTMS in fibromyalgia: a randomized trial evaluating QoL and its brain metabolic substrate. Neurology 2014;82(14):1231-1238.

[4] Braden BB, Pipe TB, Smith R, Glaspy TK, Deatherage BR, Baxter LC. Brain and behavior changes associated with an abbreviated 4-week mindfulness-based stress reduction course in back pain patients. Brain Behav 2016;6(3):e00443.

[5] Ceko M, Shir Y, Ouellet JA, Ware MA, Stone LS, Seminowicz DA. Partial recovery of abnormal insula and dorsolateral prefrontal connectivity to cognitive networks in chronic low back pain after treatment. Hum Brain Mapp 2015;36(6):2075-2092.

[6] Chen J, Wang Z, Tu Y, Liu X, Jorgenson K, Ye G, Lin C, Liu J, Park J, Lang C, Liu B, Kong J. Regional Homogeneity and Multivariate Pattern Analysis of Cervical Spondylosis Neck Pain and the Modulation Effect of Treatment. Front Neurosci 2018;12:900.

[7] Chen X, Spaeth RB, Freeman SG, Scarborough DM, Hashmi JA, Wey HY, Egorova N, Vangel M, Mao J, Wasan AD, Edwards RR, Gollub RL, Kong J. The modulation effect of longitudinal acupuncture on resting state functional connectivity in knee osteoarthritis patients. Mol Pain 2015;11:67.

[8] Chen X, Spaeth RB, Retzepi K, Ott D, Kong J. Acupuncture modulates cortical thickness and functional connectivity in knee osteoarthritis patients. Sci Rep 2014;4:6482.

[9] Chu WC, Wu JC, Yew DT, Zhang L, Shi L, Yeung DK, Wang D, Tong RK, Chan Y, Lao L, Leung PC, Berman BM, Sung JJ. Does acupuncture therapy alter activation of neural pathway for pain perception in irritable bowel syndrome?: a comparative study of true and sham acupuncture using functional magnetic resonance imaging. J Neurogastroenterol Motil 2012;18(3):305-316.

[10] Cummiford CM, Nascimento TD, Foerster BR, Clauw DJ, Zubieta JK, Harris RE, DaSilva AF. Changes in resting state functional connectivity after repetitive transcranial direct current stimulation applied to motor cortex in fibromyalgia patients. Arthritis Res Ther 2016;18:40.

[11] De Groote S, Goudman L, Peeters R, Linderoth B, Vanschuerbeek P, Sunaert S, De Jaeger M, De Smedt A, Moens M. Magnetic Resonance Imaging Exploration of the Human Brain During 10 kHz Spinal Cord Stimulation for Failed Back Surgery Syndrome: A Resting State Functional Magnetic Resonance Imaging Study. Neuromodulation 2020;23(1):46-55.

[12] Diers M, Yilmaz P, Rance M, Thieme K, Gracely RH, Rolko C, Schley MT, Kiessling U, Wang H, Flor H. Treatment-related changes in brain activation in patients with fibromyalgia syndrome. Exp Brain Res 2012;218(4):619-628.

[13] Egorova N, Gollub RL, Kong J. Repeated verum but not placebo acupuncture normalizes connectivity in brain regions dysregulated in chronic pain. Neuroimage Clin 2015;9:430-435.

[14] Ferraro S, Grazzi L, Mandelli ML, Aquino D, Di Fiore D, Usai S, Bruzzone MG, Di Salle F, Bussone G, Chiapparini L. Pain processing in medication overuse headache: a functional magnetic resonance imaging (fMRI) study. Pain Med 2012;13(2):255-262.

[15] Flodin P, Martinsen S, Mannerkorpi K, Lofgren M, Bileviciute-Ljungar I, Kosek E, Fransson P. Normalization of aberrant resting state functional connectivity in fibromyalgia patients following a three month physical exercise therapy. Neuroimage Clin 2015;9:134-139.

[16] Foell J, Bekrater-Bodmann R, Diers M, Flor H. Mirror therapy for phantom limb pain: brain changes and the role of body representation. Eur J Pain 2014;18(5):729-739.

[17] Geha P, Yang Y, Estacion M, Schulman BR, Tokuno H, Apkarian AV, Dib-Hajj SD, Waxman SG. Pharmacotherapy for Pain in a Family With Inherited Erythromelalgia Guided by Genomic Analysis and Functional Profiling. JAMA Neurol 2016;73(6):659-667.

[18] Geha PY, Baliki MN, Chialvo DR, Harden RN, Paice JA, Apkarian AV. Brain activity for spontaneous pain of postherpetic neuralgia and its modulation by lidocaine patch therapy. Pain 2007;128(1-2):88-100.

[19] Geha PY, Baliki MN, Wang X, Harden RN, Paice JA, Apkarian AV. Brain dynamics for perception of tactile allodynia (touch-induced pain) in postherpetic neuralgia. Pain 2008;138(3):641-656.

[20] Gray MA, Chao CY, Staudacher HM, Kolosky NA, Talley NJ, Holtmann G. Anti-TNFalpha therapy in IBD alters brain activity reflecting visceral sensory function and cognitive-affective biases. PLoS One 2018;13(3):e0193542.

[21] Grazzi L, Chiapparini L, Ferraro S, Usai S, Andrasik F, Mandelli ML, Bruzzone MG, Bussone G. Chronic migraine with medication overuse pre-post withdrawal of symptomatic medication: clinical results and FMRI correlations. Headache 2010;50(6):998-1004.

[22] Gustin SM, Schwarz A, Birbaumer N, Sines N, Schmidt AC, Veit R, Larbig W, Flor H, Lotze M. NMDA-receptor antagonist and morphine decrease CRPS-pain and cerebral pain representation. Pain 2010;151(1):69-76.

[23] Harris RE, Napadow V, Huggins JP, Pauer L, Kim J, Hampson J, Sundgren PC, Foerster B, Petrou M, Schmidt-Wilcke T, Clauw DJ. Pregabalin rectifies aberrant brain chemistry, connectivity, and functional response in chronic pain patients. Anesthesiology 2013;119(6):1453-1464.

[24] Harris RE, Sundgren PC, Pang Y, Hsu M, Petrou M, Kim SH, McLean SA, Gracely RH, Clauw DJ. Dynamic levels of glutamate within the insula are associated with improvements in multiple pain domains in fibromyalgia. Arthritis Rheum 2008;58(3):903-907.

[25] Harris RE, Zubieta JK, Scott DJ, Napadow V, Gracely RH, Clauw DJ. Traditional Chinese acupuncture and placebo (sham) acupuncture are differentiated by their effects on mu-opioid receptors (MORs). Neuroimage 2009;47(3):1077-1085.

[26] Harte SE, Ichesco E, Hampson JP, Peltier SJ, Schmidt-Wilcke T, Clauw DJ, Harris RE. Pharmacologic attenuation of cross-modal sensory augmentation within the chronic pain insula. Pain 2016;157(9):1933-1945.

[27] Hashmi JA, Baliki MN, Huang L, Parks EL, Chanda ML, Schnitzer T, Apkarian AV. Lidocaine patch (5%) is no more potent than placebo in treating chronic back pain when tested in a randomised double blind placebo controlled brain imaging study. Mol Pain 2012;8:29.

[28] Hashmi JA, Baria AT, Baliki MN, Huang L, Schnitzer TJ, Apkarian AV. Brain networks predicting placebo analgesia in a clinical trial for chronic back pain. Pain 2012;153(12):2393-2402.

[29] Jensen KB, Kosek E, Wicksell R, Kemani M, Olsson G, Merle JV, Kadetoff D, Ingvar M. Cognitive Behavioral Therapy increases pain-evoked activation of the prefrontal cortex in patients with fibromyalgia. Pain 2012;153(7):1495-1503.

[30] Jensen KB, Petzke F, Carville S, Choy E, Fransson P, Gracely RH, Vitton O, Marcus H, Williams SC, Ingvar M, Kosek E. Segregating the cerebral mechanisms of antidepressants and placebo in fibromyalgia. J Pain 2014;15(12):1328-1337.

[31] Kim SH, Lee Y, Lee S, Mun CW. Evaluation of the effectiveness of pregabalin in alleviating pain associated with fibromyalgia: using functional magnetic resonance imaging study. PLoS One 2013;8(9):e74099.

[32] Kishima H, Saitoh Y, Oshino S, Hosomi K, Ali M, Maruo T, Hirata M, Goto T, Yanagisawa T, Sumitani M, Osaki Y, Hatazawa J, Yoshimine T. Modulation of neuronal activity after spinal cord stimulation for neuropathic pain; H(2)15O PET study. Neuroimage 2010;49(3):2564-2569.

[33] Koeppe C, Schneider C, Thieme K, Mense S, Stratz T, Muller W, Flor H. The influence of the 5-HT3 receptor antagonist tropisetron on pain in fibromyalgia: a functional magnetic resonance imaging pilot study. Scand J Rheumatol Suppl 2004;119:24-27.

[34] Kong J, Wang Z, Leiser J, Minicucci D, Edwards R, Kirsch I, Wasan AD, Lang C, Gerber J, Yu S, Napadow V, Kaptchuk TJ, Gollub RL. Enhancing treatment of osteoarthritis knee pain by boosting expectancy: A functional neuroimaging study. Neuroimage Clin 2018;18:325-334.

[35] Krebs K, Rorden C, Androulakis XM. Resting State Functional Connectivity After Sphenopalatine Ganglion Blocks in Chronic Migraine With Medication Overuse Headache: A Pilot Longitudinal fMRI Study. Headache 2018;58(5):732-743.

[36] Lackner JM, Lou Coad M, Mertz HR, Wack DS, Katz LA, Krasner SS, Firth R, Mahl TC, Lockwood AH. Cognitive therapy for irritable bowel syndrome is associated with reduced limbic activity, GI symptoms, and anxiety. Behav Res Ther 2006;44(5):621-638.

[37] Lazaridou A, Kim J, Cahalan CM, Loggia ML, Franceschelli O, Berna C, Schur P, Napadow V, Edwards RR. Effects of Cognitive-Behavioral Therapy (CBT) on Brain Connectivity Supporting Catastrophizing in Fibromyalgia. Clin J Pain 2017;33(3):215-221.

[38] Li J, Zhang JH, Yi T, Tang WJ, Wang SW, Dong JC. Acupuncture treatment of chronic low back pain reverses an abnormal brain default mode network in correlation with clinical pain relief. Acupunct Med 2014;32(2):102-108.

[39] Li K, Zhang Y, Ning Y, Zhang H, Liu H, Fu C, Ren Y, Zou Y. The effects of acupuncture treatment on the right frontoparietal network in migraine without aura patients. J Headache Pain 2015;16:518.

[40] Li Z, Liu M, Lan L, Zeng F, Makris N, Liang Y, Guo T, Wu F, Gao Y, Dong M, Yang J, Li Y, Gong Q, Liang F, Kong J. Altered periaqueductal gray resting state functional connectivity in migraine and the modulation effect of treatment. Sci Rep 2016;6:20298.

[41] Li Z, Zeng F, Yin T, Lan L, Makris N, Jorgenson K, Guo T, Wu F, Gao Y, Dong M, Liu M, Yang J, Li Y, Gong Q, Liang F, Kong J. Acupuncture modulates the abnormal brainstem activity in migraine without aura patients. Neuroimage Clin 2017;15:367-375.

[42] Lieberman MD, Jarcho JM, Berman S, Naliboff BD, Suyenobu BY, Mandelkern M, Mayer EA. The neural correlates of placebo effects: a disruption account. Neuroimage 2004;22(1):447-455.

[43] Linnman C, Catana C, Svardsudd K, Appel L, Engler H, Langstrom B, Sorensen J, Furmark T, Fredrikson M, Borsook D, Peterson M. Decreased Brain Neurokinin-1 Receptor Availability in Chronic Tennis Elbow. PLoS One 2016;11(9):e0161563.

[44] Lowen MB, Mayer EA, Sjoberg M, Tillisch K, Naliboff B, Labus J, Lundberg P, Strom M, Engstrom M, Walter SA. Effect of hypnotherapy and educational intervention on brain response to visceral stimulus in the irritable bowel syndrome. Aliment Pharmacol Ther 2013;37(12):1184-1197.

[45] Maarrawi J, Peyron R, Mertens P, Costes N, Magnin M, Sindou M, Laurent B, Garcia-Larrea L. Motor cortex stimulation for pain control induces changes in the endogenous opioid system. Neurology 2007;69(9):827-834.

[46] Maeda Y, Kim H, Kettner N, Kim J, Cina S, Malatesta C, Gerber J, McManus C, Ong-Sutherland R, Mezzacappa P, Libby A, Mawla I, Morse LR, Kaptchuk TJ, Audette J, Napadow V. Rewiring the primary somatosensory cortex in carpal tunnel syndrome with acupuncture. Brain 2017;140(4):914-927.

[47] Magis D, Bruno MA, Fumal A, Gerardy PY, Hustinx R, Laureys S, Schoenen J. Central modulation in cluster headache patients treated with occipital nerve stimulation: an FDG-PET study. BMC Neurol 2011;11:25.

[48] Mayer EA, Berman S, Derbyshire SW, Suyenobu B, Chang L, Fitzgerald L, Mandelkern M, Hamm L, Vogt B, Naliboff BD. The effect of the 5-HT3 receptor antagonist, alosetron, on brain responses to visceral stimulation in irritable bowel syndrome patients. Aliment Pharmacol Ther 2002;16(7):1357-1366.

[49] Micalos PS, Korgaonkar MS, Drinkwater EJ, Cannon J, Marino FE. Cerebral responses to innocuous somatic pressure stimulation following aerobic exercise rehabilitation in chronic pain patients: a functional magnetic resonance imaging study. Int J Gen Med 2014;7:425-432.

[50] Napadow V, Kettner N, Liu J, Li M, Kwong KK, Vangel M, Makris N, Audette J, Hui KK. Hypothalamus and amygdala response to acupuncture stimuli in Carpal Tunnel Syndrome. Pain 2007;130(3):254-266.

[51] Napadow V, Kim J, Clauw DJ, Harris RE. Decreased intrinsic brain connectivity is associated with reduced clinical pain in fibromyalgia. Arthritis Rheum 2012;64(7):2398-2403.

[52] Neeb L, Bayer A, Bayer KE, Farmer A, Fiebach JB, Siegmund B, Volz MS. Transcranial direct current stimulation in inflammatory bowel disease patients modifies resting-state functional connectivity: A RCT. Brain Stimul 2019;12(4):978-980.

[53] Ohn SH, Chang WH, Park CH, Kim ST, Lee JI, Pascual-Leone A, Kim YH. Neural correlates of the antinociceptive effects of repetitive transcranial magnetic stimulation on central pain after stroke. Neurorehabil Neural Repair 2012;26(4):344-352.

[54] Parks EL, Geha PY, Baliki MN, Katz J, Schnitzer TJ, Apkarian AV. Brain activity for chronic knee osteoarthritis: dissociating evoked pain from spontaneous pain. Eur J Pain 2011;15(8):843 e841-814.

[55] Petzke F, Jensen KB, Kosek E, Choy E, Carville S, Fransson P, Williams SCR, Marcus H, Mainguy Y, Ingvar M, Gracely RH. Using fMRI to evaluate the effects of milnacipran on central pain processing in patients with fibromyalgia. Scand J Pain 2013;4(2):65-74.

[56] Pinto-Sanchez MI, Hall GB, Ghajar K, Nardelli A, Bolino C, Lau JT, Martin FP, Cominetti O, Welsh C, Rieder A, Traynor J, Gregory C, De Palma G, Pigrau M, Ford AC, Macri J, Berger B, Bergonzelli G, Surette MG, Collins SM, Moayyedi P, Bercik P. Probiotic Bifidobacterium longum NCC3001 Reduces Depression Scores and Alters Brain Activity: A Pilot Study in Patients With Irritable Bowel Syndrome. Gastroenterology 2017;153(2):448-459 e448.

[57] Privitera R, Birch R, Sinisi M, Mihaylov IR, Leech R, Anand P. Capsaicin 8% patch treatment for amputation stump and phantom limb pain: a clinical and functional MRI study. J Pain Res 2017;10:1623-1634.

[58] Rogachov A, Bhatia A, Cheng JC, Bosma RL, Kim JA, Osborne NR, Hemington KS, Venkatraghavan L, Davis KD. Plasticity in the dynamic pain connectome associated with ketamine-induced neuropathic pain relief. Pain 2019;160(7):1670-1679.

[59] Sanders D, Krause K, O'Muircheartaigh J, Thacker MA, Huggins JP, Vennart W, Massat NJ, Choy E, Williams SC, Howard MA. Pharmacologic modulation of hand pain in osteoarthritis: a double-blind placebo-controlled functional magnetic resonance imaging study using naproxen. Arthritis Rheumatol 2015;67(3):741-751.

[60] Sawaddiruk P, Apaijai N, Paiboonworachat S, Kaewchur T, Kasitanon N, Jaiwongkam T, Kerdphoo S, Chattipakorn N, Chattipakorn SC. Coenzyme Q10 supplementation alleviates pain in pregabalin-treated fibromyalgia patients via reducing brain activity and mitochondrial dysfunction. Free Radic Res 2019;53(8):901-909.

[61] Seminowicz DA, Wideman TH, Naso L, Hatami-Khoroushahi Z, Fallatah S, Ware MA, Jarzem P, Bushnell MC, Shir Y, Ouellet JA, Stone LS. Effective treatment of chronic low back pain in humans reverses abnormal brain anatomy and function. J Neurosci 2011;31(20):7540-7550.

[62] Shpaner M, Kelly C, Lieberman G, Perelman H, Davis M, Keefe FJ, Naylor MR. Unlearning chronic pain: A randomized controlled trial to investigate changes in intrinsic brain connectivity following Cognitive Behavioral Therapy. Neuroimage Clin 2014;5:365-376.

[63] Smallwood RF, Potter JS, Robin DA. Neurophysiological mechanisms in acceptance and commitment therapy in opioid-addicted patients with chronic pain. Psychiatry Res Neuroimaging 2016;250:12-14.

[64] Taylor AG, Anderson JG, Riedel SL, Lewis JE, Bourguignon C. A randomized, controlled, double-blind pilot study of the effects of cranial electrical stimulation on activity in brain pain processing regions in individuals with fibromyalgia. Explore (NY) 2013;9(1):32-40.

[65] Tetreault P, Baliki MN, Baria AT, Bauer WR, Schnitzer TJ, Apkarian AV. Inferring distinct mechanisms in the absence of subjective differences: Placebo and centrally acting analgesic underlie unique brain adaptations. Hum Brain Mapp 2018;39(5):2210-2223.

[66] Tillisch K, Labus J, Nam B, Bueller J, Smith S, Suyenobu B, Siffert J, McKelvy J, Naliboff B, Mayer E. Neurokinin-1-receptor antagonism decreases anxiety and emotional arousal circuit response to noxious visceral distension in women with irritable bowel syndrome: a pilot study. Aliment Pharmacol Ther 2012;35(3):360-367.

[67] Timmers I, de Jong JR, Goossens M, Verbunt JA, Smeets RJ, Kaas AL. Exposure in vivo Induced Changes in Neural Circuitry for Pain-Related Fear: A Longitudinal fMRI Study in Chronic Low Back Pain. Front Neurosci 2019;13:970.

[68] Walitt B, Roebuck-Spencer T, Esposito G, Atkins F, Bleiberg J, Foster G, Weinstein A. The effects of multidisciplinary therapy on positron emission tomography of the brain in fibromyalgia: a pilot study. Rheumatol Int 2007;27(11):1019-1024.

[69] Yoon EJ, Kim YK, Kim HR, Kim SE, Lee Y, Shin HI. Transcranial direct current stimulation to lessen neuropathic pain after spinal cord injury: a mechanistic PET study. Neurorehabil Neural Repair 2014;28(3):250-259.

[70] Yoshino A, Okamoto Y, Okada G, Takamura M, Ichikawa N, Shibasaki C, Yokoyama S, Doi M, Jinnin R, Yamashita H, Horikoshi M, Yamawaki S. Changes in resting-state brain networks after cognitive-behavioral therapy for chronic pain. Psychol Med 2018;48(7):1148-1156.

[71] Zhang Y, Li KS, Liu HW, Fu CH, Chen S, Tan ZJ, Ren Y. Acupuncture treatment modulates the resting-state functional connectivity of brain regions in migraine patients without aura. Chin J Integr Med 2016;22(4):293-301.

[72] Zhao JM, Lu JH, Yin XJ, Chen XK, Chen YH, Tang WJ, Jin XM, Wu LY, Bao CH, Wu HG, Shi Y. Comparison of electroacupuncture and moxibustion on brain-gut function in patients with diarrhea-predominant irritable bowel syndrome: A randomized controlled trial. Chin J Integr Med 2015;21(11):855-865.

[73] Zhao JM, Lu JH, Yin XJ, Wu LY, Bao CH, Chen XK, Chen YH, Tang WJ, Jin XM, Wu HG, Shi Y. Comparison of Electroacupuncture and Mild-Warm Moxibustion on Brain-Gut Function in Patients with Constipation-Predominant Irritable Bowel Syndrome: A Randomized Controlled Trial. Chin J Integr Med 2018;24(5):328-335.

[74] Zhu Y, Wu Z, Ma X, Liu H, Bao C, Yang L, Cui Y, Zhou C, Wang X, Wang Y, Zhang Z, Zhang H, Jia H, Wu H. Brain regions involved in moxibustion-induced analgesia in irritable bowel syndrome with diarrhea: a functional magnetic resonance imaging study. BMC Complement Altern Med 2014;14:500.

[75] Zou Y, Tang W, Li X, Xu M, Li J. Acupuncture Reversible Effects on Altered Default Mode Network of Chronic Migraine Accompanied with Clinical Symptom Relief. Neural Plast 2019;2019:5047463.
